# Mucoadhesive Nanoemulsions for Nose-to-Brain Delivery of Dimethyl Fumarate: Development, Characterization and Proof-of-Concept In Vivo Evaluation

**DOI:** 10.3390/pharmaceutics18091168

**Published:** 2026-09-16

**Authors:** Eleonora Sofia Cama, Giada Botti, Sara Perteghella, Laura Catenacci, Fahad Khan Tareen, Sarah Beggiato, Maria Cristina Bonferoni, Luca Ferraro, Milena Sorrenti, Alessandro Dalpiaz

**Affiliations:** 1 Department of Drug Sciences, University of Pavia, Viale Taramelli, 12, 27100 Pavia, Italy; eleonorasofia.cama01@universitadipavia.it (E.S.C.); sara.perteghella@unipv.it (S.P.); fahadkhan.tareen01@universitadipavia.it (F.K.T.); mariacristina.bonferoni@unipv.it (M.C.B.); milena.sorrenti@unipv.it (M.S.); 2 Department of Chemical, Pharmaceutical and Agricultural Sciences, University of Ferrara, Via Fossato di Mortara 19, 44121 Ferrara, Italy; bttgdi@unife.it (G.B.); dla@unife.it (A.D.); 3 Department of Life Sciences and Biotechnology, University of Ferrara, Via Borsari 46, 44121 Ferrara, Italy; bggsrh@unife.it (S.B.); frl@unife.it (L.F.)

**Keywords:** nanoemulsion, dimethyl fumarate, nose-to-brain drug delivery, geraniol, multiple sclerosis, chitosan oleate

## Abstract

**Background:** Dimethyl fumarate (DMF) is an approved therapeutic agent for the treatment of multiple sclerosis, known for its anti-inflammatory and neuroprotective properties. However, its oral administration is often accompanied by gastrointestinal side effects, limiting its therapeutic potential. In the present study, a novel nanoemulsion (NE) formulation was developed and characterized with the aim of enhancing DMF exposure in the cerebrospinal fluid (CSF) following intranasal administration. **Methods:** The formulation was prepared via a self-emulsification method using geraniol (GER) as the oil phase, selected for its antioxidant properties, and chitosan oleate as a mucoadhesive stabilizer and absorption promoter. Physicochemical characterization, in vitro release, RPMI 2650 cell cytotoxicity, and permeability were evaluated. Finally, an optimized NE (1.08 ± 0.02 mg/mL DMF and 0.28 ± 0.01 mg/mL GER) was administered intranasally to adult male Sprague–Dawley rats to assess DMF and GER exposure in the CSF. **Results:** The NEs exhibited optimal physicochemical properties: a mean particle size of approximately 170 nm, a polydispersity index below 0.3, and a positive zeta potential (above 20 mV). Spectroscopic and thermal analyses confirmed GER and DMF compatibility and a reciprocal enhancement of stability. In vitro drug release studies revealed a fast release profile, with 81% of DMF released within 2 h, aligning with the rapid absorption expected from nasal administration. Cytotoxicity assays showed high biocompatibility (>86% viability up to 50 µM DMF), while permeability studies demonstrated significant absorption, with 60% of DMF and 90% of GER absorbed within 2–3 h, favored by smaller droplet size. In rats, intranasal delivery achieved maximum CSF concentrations of ~8 µg/mL for DMF and ~1.5 µg/mL for GER within 2 h. **Conclusions:** These findings provide pharmacokinetic proof-of-concept supporting further investigation of this intranasal formulation for CNS delivery of DMF.

## 1. Introduction

Multiple sclerosis (MS) is a chronic, immune-mediated disease of the central nervous system (CNS), characterized by inflammatory demyelination and progressive neuroaxonal degeneration, ultimately leading to cognitive, sensory, and motor impairment [1,2,3]. Although its exact etiology remains incompletely understood, neuroinflammation and oxidative stress are recognized as major contributors to disease progression [4,5]. Accordingly, several therapeutic strategies currently employed in MS aim to modulate peripheral immune responses while limiting CNS damage and slowing disease progression [6].

Dimethyl fumarate (DMF), a fumaric acid ester approved by the EMA (Tecfidera^TM^) for the treatment of relapsing-remitting MS, is among the most widely used disease-modifying therapies for this purpose. Besides its established clinical efficacy, DMF exhibits potent anti-inflammatory, antioxidant, and neuroprotective properties mainly associated with activation of the nuclear factor erythroid 2-related factor 2 (Nrf2) pathway and the subsequent induction of cytoprotective antioxidant responses [7,8]. These properties have attracted considerable interest in the development of novel strategies aimed at maximizing its therapeutic potential within the CNS.

Following oral administration, however, DMF undergoes extensive and rapid hydrolysis to monomethyl fumarate (MMF), which becomes the predominant species in both humans and animals. Consequently, no measurable plasma concentrations of the parent DMF can generally be detected after oral administration [9,10,11]. MMF is widely regarded as the main active metabolite responsible for the therapeutic effects of DMF [10,12]. Recent studies have further demonstrated that DMF is rapidly converted to MMF even following intravenous administration in rats [13]. Moreover, it is known that only ~5% of administered DMF can be recovered in the bloodstream as MMF after both intravenous and oral administrations [13,14].

MMF, which has itself been approved by the FDA (Bafiertam^TM^) for relapsing MS, is known to cross the blood–brain barrier (BBB), although CNS concentrations remain substantially lower than those measured in the systemic circulation [13,15]. Nevertheless, increasing evidence suggests that the parent drug may exert pharmacological activities distinct from those of its metabolite. In particular, DMF displays greater membrane permeability, broader intracellular reactivity, and stronger neuroprotective and immunomodulatory effects than MMF in several in vitro models, including a greater ability to suppress the production of pro-inflammatory cytokines [16]. Furthermore, unlike MMF, whose intracellular activity largely depends on receptor-mediated mechanisms, DMF readily penetrates cellular membranes and interacts with multiple intracellular targets [12,16,17,18,19]. These observations suggest that increasing direct CNS exposure to DMF may provide additional therapeutic benefits beyond those achievable through systemic exposure to MMF alone. Another important limitation of DMF therapy is the occurrence of gastrointestinal adverse effects, which frequently reduce patient compliance and may compromise long-term treatment efficacy [20]. Alternative delivery approaches capable of enhancing CNS exposure while minimizing systemic and gastrointestinal side effects are therefore highly desirable. Among these approaches, intranasal administration has emerged as a promising non-invasive strategy for CNS drug delivery. By exploiting the olfactory and trigeminal pathways, drugs may bypass the BBB and reach the CNS through direct nose-to-brain transport mechanisms, including delivery to the cerebrospinal fluid (CSF) via permeation across the olfactory mucosa and neuronal transport through intracellular and extracellular routes [21,22,23,24]. In addition to its potential for CNS targeting, the nasal route offers several practical advantages, including rapid absorption, ease of administration, and avoidance of first-pass metabolism [25]. These characteristics make intranasal delivery particularly attractive for DMF, whose therapeutic potential may be limited by rapid systemic biotransformation. However, successful nose-to-brain delivery requires formulations capable of enhancing drug solubility and stability, promoting efficient permeation across the nasal mucosa, and ensuring appropriate release profiles to maximize therapeutic efficacy [26]. Several formulation approaches have recently been investigated to overcome the poor aqueous solubility and limited stability of DMF, including lipid-based carriers and nanotechnological delivery systems [13,27,28,29,30].

Among these, nanoemulsions (NEs) have attracted considerable interest because of their ability to solubilize lipophilic compounds, improve formulation stability, and enhance drug interaction with biological membranes. NEs are colloidal systems composed of oil and aqueous phases stabilized by surfactants, typically exhibiting droplet sizes in the range of 20–500 nm [31,32]. Due to their small droplet size, NEs provide a large interfacial area that can facilitate mucosal interaction, drug absorption, and rapid release, making them particularly suitable for intranasal drug delivery applications [33,34,35,36,37,38].

Geraniol (GER), a naturally occurring monoterpene alcohol widely present in essential oils, has recently attracted attention for its antioxidant, anti-inflammatory, and neuroprotective properties [39,40,41,42,43]. Previous studies demonstrated that incorporating GER into colloidal formulations enhances its permeation across biological membranes [39,44], thereby potentially improving its pharmacological efficacy. Moreover, nasal formulations based on cyclodextrin complexes and nanoparticulate systems have already been proposed to improve its brain delivery [42,43]. Beyond its pharmacological activity, GER represents a suitable lipophilic phase for the incorporation of DMF, potentially contributing both to drug solubilization and to the therapeutic profile of the final formulation. The combination of DMF and GER may therefore represent an innovative strategy to simultaneously enhance drug delivery and provide complementary neuroprotective effects.

Chitosan, a biocompatible and biodegradable polysaccharide, is widely used in drug delivery due to its mucoadhesive, antimicrobial, and permeability-enhancing properties. Its cationic nature promotes interaction with negatively charged biological membranes and transient opening of tight junctions, thereby enhancing paracellular transport and mucosal retention, improving paracellular permeability and drug retention at mucosal sites [45,46]. Among its derivatives, chitosan oleate (CSOA), an amphiphilic ionic complex of chitosan and oleic acid, has previously demonstrated suitability as both a self-assembling carrier to obtain polymeric micelles [47] and a stabilizer for NE systems [33]. In the first case, CSOA is exploited for its auto-assembling behavior, enhancing hydrophobic drug loading in core hydrophobic domains. In NEs, CSOA contributes to stabilization, enhancing the physical stability of the dispersed phase, while preserving the mucoadhesive and penetration-enhancing properties of chitosan, potentially improving nasal absorption through the combined effects of both polysaccharide and oleic acid [44]. Based on these considerations, the aim of the present study was to develop and characterize a GER-based NE containing DMF for intranasal administration and potential nose-to-brain delivery. The formulation was stabilized using CSOA and characterized in terms of physicochemical properties, drug loading, stability, release profile, antioxidant activity, and permeation across RPMI 2650 nasal epithelial cells. Finally, based on the results of the first phase of pharmaceutical development, a NE formulation was prepared for in vivo administration, and an in vivo proof of concept study in rats was performed to evaluate the ability of the formulation to promote the delivery of DMF and GER to the CNS following intranasal administration.

## 2. Materials and Methods

### 2.1. Materials

Dimethyl fumarate (DMF, 99%) was purchased from AK Scientific Inc. (Union City, CA, USA). Monomethyl fumarate (MMF, 97%), geraniol (GER, 98%), ferulic acid (Fer), carbazole, carbamazepin (CBZ), cellulose membrane (cut-off 14 kDa, 1.96 mL/cm), ascorbic acid (AA, 2,2-diphenyl-1-picrylhydrazyl (DPPH), 3-(4,5-dimethylthiazol-2-yl)-2,5-diphenyltetrazolium bromide) (MTT) and bovine serum albumin (BSA) were purchased from Sigma Aldrich, Co. (St. Louis, MO, USA). Chitosan base (CS, batch number: STBF8219V, deacetylation degree: 75–85%, molecular weight about 187 kDa, viscosity: 20–300 mPas) was acquired from Sigma-Aldrich (Milan, Italy), while low-molecular-weight chitosan-HCl (CS-HCl, batch number: 312-090216-01, molecular weight: about 165 kDa, viscosity < 20 mPas) was acquired from Heppe Medical Chitosan GmbH (Halle, Germany). Both chitosans are obtained by the deacetylation of chitin. The chitin for HMC’s chitosan comes mainly from crustacea (*Chionoecetes opilio*).

Oleic acid was purchased from Fluka Analytical (Neu-Ulm, Germany). Acetic acid glacial, formic acid (99%), ethanol (96°), methanol (99%) and acetonitrile (99%) were acquired from Carlo Erba Reagents (Val-de-Reuil, France).

Ultrapure water was obtained from a MilliQ^®^ apparatus (Millipore, Burlington, MA, USA).

### 2.2. Physicochemical Characterization

#### 2.2.1. Solubility Studies of Dimethyl Fumarate in Geraniol

Solubility studies of DMF in GER were carried out by preparing a supersaturated solution at 25 ± 1 °C. DMF was gradually and incrementally added to 1 mL of GER under continuous magnetic stirring, with a total of approximately 50 mg of DMF introduced. The solution was then stirred overnight at 300 rpm, until equilibrium was achieved. The resulting solution was then centrifuged at 10,000 rpm for 3 min, and the supernatant was collected, filtered and analyzed for DMF content. The solubility results were reported as mean ± standard deviation (SD).

#### 2.2.2. Fourier Transform Infrared Spectroscopy

Fourier Transform Infrared (FT-IR) spectra were recorded using a Perkin Elmer Spectrum One spectrophotometer (Perkin Elmer, Monza, Italy) equipped with an ATR accessory (MIRacle™ device, Pike Technologies, Madison, WI, USA) fitted with a zinc selenide (ZnSe) crystal. The spectra were acquired in transmittance mode, within the mid-infrared (Mid-IR) range of 650 to 4000 cm^−1^, using 64 scans at a resolution of 4 cm^−1^ at ambient conditions. Each measurement was collected at least in triplicate.

#### 2.2.3. Thermal Analysis

Differential scanning calorimetry (DSC) analyses were carried out on DMF, GER, and on their physical mixtures at different weight ratios, using a Mettler Star^e^ System (Mettler Toledo, Milan, Italy) coupled with a DSC 821^e^ Module and an Intracooler device for sub-ambient temperature analysis (Julabo FT 900, Seelbach, Germany). The instrument was calibrated with Indium as a reference standard. Samples (2–12 mg), accurately weighed with a Mettler M3 Microbalance, were hermetically sealed in aluminum crucibles (40 μL capacity) with lids. Measurements were performed under a nitrogen atmosphere (50 mL/min) at a heating rate of 10 K/min, in a temperature range of 30–350 °C.

Thermogravimetric analysis (TGA) was performed on the same samples using a Mettler Star^e^ System TGA/DSC1 (Mettler Toledo, Milan, Italy), also calibrated with Indium as a reference standard. Samples (3–20 mg) were placed in alumina crucibles (70 μL capacity) with enclosed lids. All measurements were performed at least in triplicate.

#### 2.2.4. Preparation of Chitosan Oleate

Chitosan oleate (CSOA) was prepared in 1:0.5 and 1:1 (mol/mol) ratios, following a modified protocol from previous studies [44]. Briefly, 1.5 g of chitosan base (CS) was accurately weighed using an analytical balance (ADJ 100-4, max 120 g, d = 0.1 mg, Kern & Sohn, Balingen, Germany) and added to a reaction flask with 7.5 mL of ethanol and 0.3 g of acetic acid. The mixture was stirred continuously using a magnetic stirrer (ARE Heating Magnetic Stirrer, Velp Scientifica, Usmate Velate, Italy). In a second flask, either 1.8 g or 0.9 g of oleic acid (corresponding to 1:0.5 and 1:1 chitosan-to-oleic acid ratios, respectively) were dissolved in 7.5 mL of ethanol under magnetic stirring until complete solubilization. The final solution was gradually added to the first reaction mixture and then stirred overnight. Ethanol was removed the following day using a rotary evaporator (Laborota 4000, Heidolph, Schwabach, Germany) at 45 °C and 150 rpm for approximately 20 min. To remove excess oleic acid, the resulting solid was divided into two 14 mL conical tubes, each containing 6 mL of ethanol; the tubes were centrifuged (SL8, Thermo Scientific, Waltham, MA, USA) at 3000 rpm for 15 min, and the supernatant was discarded. This washing step was repeated once more. The remaining pellet was dried in a crystallizer for 24 h, yielding the CSOA powder.

#### 2.2.5. Preparation of Blank and Dimethyl Fumarate-Loaded Nanoemulsions

In this study, both blank and DMF-loaded nanoemulsions (NEs) were prepared using two different preparative methods: phase inversion emulsification and spontaneous emulsification. All NEs were prepared with the same main components: GER as the oil phase, ultrapure water as the aqueous phase, and CSOA as the amphiphilic surfactant.

#### 2.2.6. Phase Inversion Emulsification Method

The first method used for NE preparation involves a combination of phase inversion emulsification (PI) with a high-energy method using an Ultra-Turrax T18 basic homogenizer (Janke & Kunkel, IKA Labortechnik, Staufen, Germany). A water-in-oil (W/O) emulsion was initially formed by adding CSOA at chitosan-to-oleic acid molar ratios of 1:0.5 and 1:1, at final concentrations of 2% and 3%, along with acetic acid (for chitosan solubilization), into the oil phase. The minimum volume of MilliQ^®^ ultrapure water needed to achieve a W/O dispersion was then gradually added. Emulsification was performed using the Ultra-Turrax, starting at 3600 rpm for the first 3 min while the remaining MilliQ^®^ ultrapure water was added dropwise, inducing phase inversion. The resulting emulsion was centrifuged at 7000 rpm for 3 min (Mini centrifuge LLG-uniCFUGE5, LLG Labware, Meckenheim, Germany), and subsequently sonicated (Sonic Dismembrator, Fisher Scientific, Waltham, MA, USA, 3 mm probe) for 18 min at 20% amplitude, with a pulsed cycle of 10 s on and 10 s off. To ensure a stable temperature, an ice bath was used to dissipate any heat generated during this process.

DMF-loaded NEs were prepared following the same protocol, with 5 mg of DMF added to 250 mg of the oil phase at the start of the process.

#### 2.2.7. Spontaneous Emulsification Method

In this method, 6 mg of chitosan-HCl (CS-HCl) was dissolved in 6 mL of MilliQ^®^ ultrapure water. The lipophilic phase was prepared by dissolving 8.4 mg of oleic acid (maintaining a 1.4 mg:1 mg ratio with CS-HCl) and 14.4 mg of GER (to achieve a CSOA:GER, ratio of 1:1) in the minimum ethanol volume required for complete solubilization. The organic phase was then added dropwise to the aqueous phase under continuous magnetic stirring at 500 rpm. Diffusion of the organic phase into the external aqueous phase induced spontaneous formation of an oil-in-water (O/W) NE. The emulsion was stirred overnight to allow partial ethanol evaporation, with any residual solvent removed under a nitrogen stream. The final sample underwent ultrasonication (Sonic Dismembrator, Fisher Scientific, 3 mm probe) for 15 min at 20% amplitude, using a pulsed cycle of 10 s on and 10 s off.

The DMF-loaded NE was prepared following the same procedure by adding different amounts of DMF, as indicated later in the results section, which were incorporated into the organic phase before emulsification.

#### 2.2.8. Determination of Size and Zeta Potential

The droplet size of the NEs was determined by photon correlation spectroscopy (PCS) with an N5 submicron particle analyzer (Beckman Coulter, Milan, Italy) and by Dynamic Light Scattering (DLS) with a Zetasizer Nano ZS90 (Malvern Instruments, Worcestershire, UK). PCS measurements were carried out at 25 °C with a fixed scattering angle of 90°, following appropriate sample dilution in MilliQ^®^ ultrapure water (1:10 to 1:100, *v*/*v*). The zeta potential of the NEs was measured by DLS using an Omega cuvette (DTS1070, Malvern Instruments, Worcestershire, UK) under the same temperature and detection angle conditions. All measurements were performed in triplicate, and results are reported as mean ± SD.

#### 2.2.9. Transmission Electron Microscopy

The ultrastructural characterization of unloaded and loaded NEs was carried out using transmission electron microscopy (TEM, Jeol-Jem-1200EXII, Tokyo, Japan). For sample preparation, a drop of DMF-loaded NE or its unloaded counterpart was placed on a copper grid and air-dried at 25 °C for 2 min. The grid was then stained with uranyl acetate prior to imaging.

#### 2.2.10. Stability Studies

The stability of the different NEs was evaluated by monitoring droplet size over time. Particle size was measured weekly by PCS for up to six weeks in formulations prepared by the phase inversion and spontaneous emulsification methods. NEs were aliquoted and stored under two different conditions: refrigerated storage (4 ± 1 °C) and room temperature (25 ± 1 °C).

At each time point, measurements were performed in triplicate, and results were expressed as mean ± SD.

#### 2.2.11. Quantification of Dimethyl Fumarate and Geraniol in Nanoemulsions

The samples of NEs (100 μL) were first diluted with 45 μL of acetate buffer 100 mM (pH 4) and with 855 μL of acetonitrile, the latter being added after vortexing. These samples were further diluted 1:10 using a mixture of acetonitrile:buffer acetate 95:5 (*v*/*v*) and analyzed by the high-performance liquid chromatography (HPLC) system below described, after filtration through regenerated cellulose filters (0.45 μm), for the detection and quantification of DMF and GER. For the preliminary characterization of the NEs, a specific HPLC method was adapted for the detection of both compounds in the same chromatogram [48,49].

In particular, the analyses were performed using an HPLC system (1100 Series, Agilent, Santa Clara, CA, USA) equipped with an endcapped C18 column (150 mm × 4.6 mm, 3 μm). The mobile phase consisted of H_2_O:formic acid (pH 3.0 ± 0.1; DL40GP, MemoTitrator, Mettler, Milan, Italy) and acetonitrile (60:40 *v*/*v*) with a flow rate of 0.8 mL/min. Chromatographic separations were carried out at room temperature (25 ± 1 °C) under a pressure of 112 bar. Under these conditions, the retention times were 4.8 min for DMF and 13.18 min for GER. The injection volume was 20 μL. To avoid cross-contamination, the injection needle was rinsed with acetonitrile after each sample run. Detection was performed at a wavelength of 210 nm [49,50].

Calibration curves were constructed for both analytes. For DMF, a six-point calibration curve was prepared from a 1 mg/mL stock solution in methanol diluted in the mixture of acetonitrile:buffer acetate 95:5 (*v*/*v*) to cover the concentration range of 0.2–1.8 μg/mL, with carbamazepine (CBZ) (final concentration 8 μg/mL) as an internal standard. The assay showed good linearity within this range (y = 0.855x + 0.001; R^2^ = 0.9977).

Similarly, for GER, a six-point calibration curve was generated from a 1 mg/mL stock solution in methanol diluted in the mixture of acetonitrile:buffer acetate 95:5 (*v*/*v*) to cover a range of concentrations of 2–50 μg/mL. Each sample also contained CBZ (8 μg/mL) as an internal standard. Also, for GER, linearity was confirmed within the selected range (y = 0.0304x − 0.0255; R^2^ = 0.9971).

#### 2.2.12. Recovery and Loading Capacity

Recovery% (*Rec*%) and drug loading (*DL*%) were determined on the loaded formulation by HPLC analysis using the conditions described above, using the following Equations (1) and (2) [51]:

(1) $$Rec\%=\frac{L_{DMF}\;}{T_{DMF}}\times 100$$

(2) $$DL\%=\frac{L_{DMF}}{T_{formulation}}\times 100$$

where *L_DMF_* is the amount of DMF loaded in the NE, *T_DMF_* is the total amount of DMF added to the formulation, and *T_formulation_* is the total weight of the formulation (CSOA, DMF and GER).

All determinations were performed in triplicate in independent experiments, and results are expressed as mean ± SD.

#### 2.2.13. In Vitro Release Studies

The in vitro release profile of the formulation containing DMF in GER was evaluated using a dialysis cellulose membrane with a molecular weight cut-off of 14 kDa. The membrane was filled with the sample and immersed in 140 mL of phosphate-buffered saline (PBS, pH 4) prepared according to the European Pharmacopoeia (Ph. Eur. 10.5 Ed.). The receiving medium was maintained at 34 ± 1 °C under constant magnetic stirring at 500 rpm. At predetermined time intervals (ranging from 5 to 240 min), 1 mL aliquots were withdrawn from the receiving medium and immediately replaced with an equal volume of fresh PBS to maintain sink conditions for both DMF and GER. Their concentrations in the release medium were determined by HPLC, as previously described. All experiments were conducted in triplicate, with results expressed as mean ± SD.

#### 2.2.14. ROS-Scavenging Activity

The reactive oxygen species (ROS)-scavenging activity of NEs was evaluated by the 2,2-diphenyl-1-picrylhydrazyl (DPPH) colorimetric assay (Sigma Aldrich, Milan, Italy). Each formulation was tested after dilution at 1:3, 1:5 and 1:10 in MilliQ^®^ ultrapure water. For each measurement, a 100 μL aliquot of the sample was mixed with 900 μL of a DPPH solution (0.0112% in 70% *v*/*v* methanol) and incubated in the dark for 1 h at room temperature (25 ± 1 °C). A negative control, consisting of a methanol–DPPH mixture without NEs, was prepared for comparison. Following incubation, the samples were centrifuged at 15,000 rpm for 10 min, and the absorbance of the supernatant was recorded at 517 nm using a microplate reader (Synergy HT, BioTek, Swindon, UK). The ROS-scavenging activity percentage was calculated using the following Equation (3):

(3) $$Absorbance\%=\frac{A_{ctrl}-A_{samp}}{A_{ctrl}}\times 100$$

where *A_ctrl_* is the absorbance of the negative control, and *A_samp_* is the absorbance of the tested sample, corrected for the corresponding blank. Ascorbic acid (3.125–100 μg/mL) was used as a positive control. All experiments were carried out at least in triplicate and the results reported as mean ± SD.

#### 2.2.15. Cell Culture

Immortalized human nasal epithelial cell line RPMI 2650, derived from an anaplastic squamous cell carcinoma of the human nasal septum, was obtained from the American Type Culture Collection (ATCC). Cells were used between passages P3 and P5 for in vitro cytotoxicity and permeability assays. The cells were maintained in T75 polystyrene culture flasks (SPL Life Sciences, Pocheon-si, South Korea) at a seeding density of 5 × 10^5^ cells/cm^2^ [52] and incubated at 37 °C with 5% CO_2_. The culture medium was refreshed every 2–3 days. Cell counts were determined using a Dual Fluorescence Cell Counter (LUNA-FL™, Logos Biosystems, Anyang-si, South Korea). Cell maintenance was performed in αMEM medium (PAN Biotech, Aidenbach, Germany), supplemented with 10% fetal bovine serum (FBS), 1% sodium pyruvate, 1% glutamine, and 1% penicillin-streptomycin-amphotericin [53].

Upon reaching 80% confluency, cells were detached with 1X Trypsin with calcium, magnesium, and phenol red (Biosigma, Cona, Italy) at 37 °C. For the cytotoxicity assay, suspended cells were then seeded at a density of 1.5 × 10^5^ cells/cm^2^ in 96-well plates, while for permeability experiments, cells were seeded at 5 × 10^5^ cells/cm^2^ in 24-well Transwell plates.

#### 2.2.16. Cytotoxicity Studies

The in vitro cytotoxicity of DMF, DMF-loaded and unloaded NEs was assessed on RPMI 2650 cells to determine their effects and the highest non-cytotoxic concentration for subsequent permeability studies. Cytotoxicity evaluation was performed using the MTT assay (3-(4,5-dimethylthiazol-2-yl)-2,5-diphenyltetrazolium bromide). Cells were seeded in 96-well plates at a density of 1.5 × 10^5^ cells/cm^2^ and cultured for approximately 6 days [54]. After incubation, the culture medium was replaced with 100 μL of the tested sample. Cells were exposed to the samples for 2, 6, 24, and 48 h. Following incubation, the samples were discarded, and 100 μL of MTT solution (0.5 mg/mL) was added to each well and incubated at 37 °C with 5% CO_2_ for 3 h. After incubation, the MTT solution was removed, and 100 μL of dimethyl sulfoxide (DMSO) was added to dissolve the formazan crystals formed. Cytotoxicity was evaluated across six increasing concentrations of DMF (5, 10, 20, 30, 50, and 100 μM), both as a free compound (stock solution prepared at a concentration of 1670 µM in methanol:culture medium 30:70 (*v*/*v*)) and formulated in NEs, all diluted in culture medium. Blank NEs were subjected to the same dilution series as DMF-loaded NEs. Untreated cells served as controls (100% metabolic activity). The optical density (OD) of the solutions was measured at 570 nm, with a reference wavelength of 670 nm, using a microplate reader (Synergy HT, Agilent Technologies, BioTek, Winooski, VT, USA) [55].

*Cell viability* (%) was calculated according to the following Equation (4):

(4) $$Cell\;viability\%=\frac{{OD}_{sample}}{{OD}_{control}}\times 100$$

All experiments were performed in triplicate and the results expressed as mean ± SD.

#### 2.2.17. Permeability Studies

Permeability assessments were performed on both DMF alone and DMF-loaded NEs prepared by the spontaneous emulsification method (containing 10% DMF in the oil phase) at the concentration selected by the MMT assay (50 μM). DMF alone was dissolved in a mixture of methanol:culture medium (volume ratio 30:70) at a concentration of 1670 µM; the stock solution was then diluted in culture medium (DMF final concentration: 30 µM, methanol final concentration: 0.5%). To assess the relevance of NE particle size, permeability was evaluated under the same conditions also on the NE prepared by the high-energy method. RPMI 2650 cells were seeded at a density of 5 × 10^5^ cells/cm^2^ [52] in 24-well Corning Costar Transwell plates (Sigma Aldrich, Milan, Italy, Lot#1701) with polycarbonate membranes of 0.4 μm pore size. The cells were cultured at 37 °C with 5% CO_2_ for 3 weeks, with the medium replaced every 2–3 days. During the first 8 days, cells were cultured under liquid–liquid conditions, with medium present in both the apical (donor) and basal (acceptor) compartments of the Transwells. Thereafter, the apical medium was removed, and cells were maintained at the air–liquid interface (ALI), as previously reported by Kreft et al. [53].

The transepithelial electrical resistance (TEER) was monitored weekly using a Millicell ERS-2 voltmeter (Millicell, Merck Millipore, Darmstadt, Germany). Permeability testing was conducted 21 days post-seeding, when the TEER value reached 57 ± 2 Ω·cm^2^ [54], consistent with the expected range for human nasal mucosal cells (40–120 Ω·cm^2^) [56,57].

For permeability evaluation, the culture medium from both compartments was removed, and wells were washed twice with PBS. Subsequently, 600 μL of phenol red-free, FBS-free αMEM medium was added to the basal compartment, while 200 μL of each test sample was introduced into the apical compartment. At designated time points (30, 60, 120, and 180 min), 300 μL of medium was withdrawn from the basal compartment for DMF quantification and immediately replaced with fresh medium.

The apparent permeability coefficient, *Papp* (cm s^−1^), measures drug permeability across the cell monolayer, and is calculated according to the following Equation (5):

(5) Papp=dQdtA·C0

where *dQ* (μg) is the amount of drug permeated from the apical to the basolateral side during the incubation time *t* (s), *A* is the surface area of the insert (cm^2^), and *C*_0_ is the initial concentration of DMF on the apical side (μg/mL) [54,58,59].

All collected samples were analyzed by HPLC, according to the method previously described, and the results were expressed as the mean percentage of permeated DMF ± SD for each experimental condition considered.

All cell culture procedures were performed under aseptic conditions using sterile culture materials, media, and reagents. The nanoemulsion formulations were not subjected to terminal sterilization before addition to the cultures.

#### 2.2.18. Quantification of Monomethyl Fumarate, Dimethyl Fumarate and Geraniol in the Nanoemulsions Formulated for In Vivo Studies

Samples (100 μL) of the NEs formulated for in vivo studies with a DMF theoretical concentration of 2.5 mg/mL (NE2.5) were first diluted with 45 μL of acetate buffer 100 mM (pH 4) and with 855 μL of acetonitrile, the latter being added after vortexing. These samples were further diluted 1:10 using a mixture of acetonitrile:buffer acetate 95:5 (*v*/*v*), filtered through regenerated cellulose filters (0.45 μm) and injected (10 μL) into the same HPLC systems used for the quantification of monomethyl fumarate (MMF), DMF and GER, described in the section below.

#### 2.2.19. Nasal Administration of Nanoemulsions to Rats

Adult male Sprague–Dawley rats (200–250 g; Charles River Laboratories, Calco, Italy) were used for the in vivo experiments. Animals were housed in groups of five per cage under controlled temperature and relative humidity conditions and a regular 12 h light/dark cycle, with food and water available ad libitum and environmental enrichment. Following arrival at the animal facility, animals were allowed to acclimatize for at least one week before the experimental procedures. Animals were monitored daily throughout the study, and a body condition score was recorded to assess their general health status. Humane endpoints included a body weight loss greater than 20% and signs of marked distress, including persistent isolation in a corner of the cage or an abnormal flattened posture. Only clinically healthy animals were included in the study.

A total of eight animals were included and allocated to two independent analytical cohorts (*n* = 4 each): one for the pharmacokinetic analysis of DMF/MMF and the other for the pharmacokinetic analysis of GER. The individual animal represented the experimental unit. Given the exploratory proof-of-concept nature of the in vivo pharmacokinetic study, no formal a priori sample-size calculation was performed. The sample size was selected based on previous pharmacokinetic studies performed by our group using comparable experimental approaches, while minimizing animal use in accordance with the principle of reduction. No additional predefined exclusion criteria were applied, and no animals or experimental data were excluded from the analyses. No formal randomization procedure was used for allocation to the two analytical cohorts, since both cohorts received the same intranasal treatment and differed only in the analytes subsequently quantified. The investigator performing the HPLC analyses was blinded to the experimental allocation of the samples.

Rats were anesthetized with isoflurane (2–5%) and placed in the supine position. Each animal received 50 μL of NE2.5 into each nostril (total administration volume, 100 μL), corresponding to approximately 0.5 mg/kg DMF and 0.14 mg/kg GER. Intranasal administration was performed using a semiautomatic pipette connected to a short polyethylene tube inserted approximately 0.6 cm into the nostril. NE2.5 was freshly prepared and handled under controlled laboratory conditions prior to administration. The formulation was not subjected to terminal sterilization. Given the single-dose design and the short pharmacokinetic observation period (150 min), no terminal sterilization step was included in the experimental protocol. During the experimental procedures, animals were continuously observed for changes in respiratory pattern as indicators of adverse reactions. No adverse events related to intranasal administration or sampling procedures were observed, and none of the animals reached the predefined humane endpoints.

At predetermined time points, CSF (30 μL) and blood (100 μL) samples were collected. CSF collection was performed according to the cisternal puncture method described by van den Berg et al. [60], requiring a single needle insertion and allowing the collection of virtually blood-free serial CSF samples (30–40 μL) [61]. No more than 120 μL of CSF per rat (i.e., four 30 μL samples per rat) was collected within 150 min. Sampling time points (*n* = 3–4 per time point) were selected taking into account the maximum of four CSF collections per animal, thereby allowing physiological restoration of CSF volume between sampling procedures.

Blood samples were serially collected from the tail vein using a butterfly needle to minimize animal stress and facilitate repeated sampling. CSF samples (10 μL) were immediately injected into the appropriate HPLC system (see below) for compound quantification.

For DMF and MMF quantification, blood samples from the DMF/MMF cohort (*n* = 4) were immediately mixed with 250 μL of ice-cold acetonitrile, followed by the addition of 50 μL of 100 μM Fer (dissolved in water/acetonitrile, 25:75, *v*/*v*) as the internal standard. Samples were centrifuged twice at 16,000× *g* for 5 min at room temperature. DMF and MMF were quantified by injecting 10 μL of the resulting supernatant into the appropriate HPLC system (see below).

For GER quantification, blood samples from the GER cohort (*n* = 4) were immediately mixed with 250 μL of ice-cold acetonitrile, followed by the addition of 50 μL of 100 μM carbazole (dissolved in water/acetonitrile, 25:75, *v*/*v*) as the internal standard. Samples were centrifuged twice at 16,000× *g* for 5 min at room temperature. GER was quantified by injecting 10 μL of the resulting supernatant into the appropriate HPLC system (see below).

The areas under the concentration–time curves (AUCs; μg·min·mL^−1^) for DMF, MMF, and GER in blood and CSF following intranasal administration of NE2.5 were calculated using the trapezoidal method.

##### Monomethyl Fumarate and Dimethyl Fumarate Quantification by HPLC Analysis

The HPLC system used for the MMF and DMF quantification in rat blood and CSF samples was a modular chromatographic system, consisting of a pump and a diode-array detector (DAD), models LC-40D and SPD-M40 (Shimadzu, Kyoto, Japan), respectively. This system was paired with an injection valve containing a 20 μL sample loop (model 7725; Rheodyne, IDEX, Torrance, CA, USA). Data acquisition and processing were performed with LabSolutions Software, version 5.110 (Shimadzu). A 5 μm Hypersil BDS C18 column (150 mm × 4.6 mm i.d.; ThermoFisher Scientific Srl, Milan, Italy), protected by a guard column filled with the same stationary phase, was used for separations that were performed at room temperature. A water:acetonitrile 80:20 (*v*/*v*) mixture acidified with 0.1% (*v*/*v*) TFA constituted the mobile phase, which was eluted under isocratic conditions at a flow rate of 0.8 mL/min. Chromatograms were recorded at 216 nm for the quantification of MMF and DMF, and at 320 nm for Fer, used as the internal standard in the blood extraction procedures. The retention times for MMF, Fer, and DMF were 3.8, 6.4, and 8.2 min, respectively.

The chromatographic precision for each compound was obtained by repeated analysis (*n* = 6) of their 50 μM solutions (6.66 μg/mL MMF; 7.21 μg/mL DMF) in a mixture of water:acetonitrile 25:75 (*v*/*v*) and it was represented by relative standard deviation (RSD) values ranging from 0.89 to 0.96 for the tested compounds. 

The calibration of DMF and MMF in CSF was performed by using a simulation fluid, constituted by a balanced Dulbecco’s phosphate-buffered saline (DPBS) solution depleted of calcium and magnesium and supplemented with 0.45 mg/mL BSA [62,63]. The concentration range of calibration in simulated CSF was 0.3–100 μM for both MMF and DMF (0.04–13.13 μg/mL for MMF and 0.04–14.41 μg/mL for DMF) and was found to be linear (*n* = 6, r = 0.998, *p* < 0.0001). Preliminary injections of blank rat CSF and whole blood samples showed no detectable interfering peaks at the retention times of MMF, DMF and the internal standard. The analytical procedure was developed and assessed for its suitability for the present exploratory preclinical study rather than subjected to a complete regulatory bioanalytical validation. Importantly, CSF samples were analyzed immediately after collection without freezing or prolonged storage, thereby minimizing possible degradation of DMF before chromatographic analysis. To further assess the identity and chromatographic specificity of the DMF peak detected in CSF, the original HPLC-DAD data were retrospectively examined using LabSolutions software. The UV spectral profile of the chromatographic peak at the DMF retention time (8.2 min) in a representative CSF sample collected 30 min after NE2.5 administration was compared with that obtained from an authentic 100 μM DMF standard prepared in simulated CSF and analyzed under the same chromatographic conditions. In addition, spectral homogeneity across the CSF peak was evaluated by DAD peak-purity analysis using the peak-purity function of the acquisition software. The corresponding chromatograms, UV spectra, and peak-purity analysis are reported in the Supplementary Materials (Figures S1–S5).

MMF recovery from whole blood samples was approximately 84%. This value was obtained by comparing the peak areas derived from 50 μM (6.66 μg/mL) MMF blood test samples (*n* = 4) to those obtained by injecting an equivalent concentration of MMF dissolved in a water:acetonitrile 25:75 (*v*/*v*) mixture. MMF blood concentration was therefore calculated using the peak area ratio with respect to the internal standard (Fer). The MMF calibration in rat whole blood (4 °C) was obtained at six different concentrations ranging from 0.5 to 30 μM (0.07–3.99 μg/mL), producing a linear relationship (*n* = 6, r = 0.995, *p* < 0.0001).

##### Geraniol Quantification by HPLC Analysis

The HPLC system used for the GER quantification in rat blood and CSF samples was a modular chromatographic system, consisting of a pump and a variable wavelength UV-Vis detector, models LC-10 AD VD and SPD-10A VP (Shimadzu, Kyoto, Japan), respectively. Also, this system was paired with an injection valve containing a 20 μL sample loop (model 7725; Rheodyne, IDEX, Torrance, CA, USA). Data acquisition and processing were performed with CLASS-VP Software, version 7.2.1 (Shimadzu). A 5 μm Hypersil BDS C18 column (150 mm × 4.6 mm i.d.; ThermoFisher Scientific Srl, Milan, Italy), protected by a guard column filled with the same stationary phase, was used for separations that were performed at room temperature. A water:acetonitrile 53:47 (*v*/*v*) mixture constituted the mobile phase, which was eluted under isocratic conditions at a flow rate of 1.0 mL/min. Chromatograms were recorded at 210 nm for the quantification of GER and carbazole, the latter being used as the internal standard in the blood extraction procedures. The retention times for GER and carbazole were 6.7 and 9.0 min, respectively.

The chromatographic precision for GER was obtained by repeated analysis (*n* = 6) of its 20 μM solution (3.08 μg/mL) in a mixture of water:acetonitrile 25:75 (*v*/*v*) and it was represented by a relative standard deviation (RSD) value of 0.91. 

The calibration of GER in CSF was performed by using the same simulation fluid described above for the calibration of DMF and MMF. The concentration range of GER calibration in simulated CSF was 0.5–20 μM (0.077–3.086 μg/mL) and was found to be linear (*n* = 6, r = 0.999, *p* < 0.0001). Preliminary injections of blank rat CSF and whole blood samples showed no detectable interfering peaks at the retention times of GER and the internal standard. The analytical procedure was developed and assessed for its suitability for the present exploratory preclinical study rather than subjected to a complete regulatory bioanalytical validation.

GER recovery from whole blood samples was approximately 70%. This value was obtained by comparing the peak areas derived from 10 μM (1.543 μg/mL) GER blood test samples *(n* = 4) to those obtained by injecting an equivalent concentration of GER dissolved in a water:acetonitrile 25:75 (*v*/*v*) mixture. GER blood concentration was therefore calculated using the peak area ratio with respect to the internal standard (carbazole). The GER calibration in rat whole blood (4 °C) was obtained at six different concentrations ranging from 0.5 to 30 μM (0.077–4.629 μg/mL), producing a linear relationship (*n* = 6, r = 0.997, *p* < 0.0001).

Selectivity of the analytical method was additionally supported by the markedly different chromatographic behavior expected for the low-molecular-weight analytes and the highly lipophilic [64,65] or polymeric components [66] of the nanoemulsion.

#### 2.2.20. Statistical Analysis

Statistical analysis was performed using Stat Graphics Centurion 18.0 (Statgraphics Technologies, Inc., Rockville, MD, USA).

Results are expressed as mean ± SD. Comparisons between two groups were performed using Student’s *t*-test, whereas comparisons among more than two groups were carried out by one-way analysis of variance (ANOVA) followed by Fisher’s least significant difference (LSD) post hoc test. One-way ANOVA was applied to evaluate the effects of formulation variables on NEs size, polydispersity index, recovery, drug loading, release behavior, antioxidant activity, permeability, and apparent permeability coefficients (Papp). Student’s *t*-test was used for direct comparisons between two formulations when appropriate.

For the in vivo pharmacokinetic studies, concentrations of DMF, MMF and GER in blood and CSF are reported as mean ± SD. Areas under the concentration–time curves (AUC) were calculated using the trapezoidal rule with GraphPad Prism version 7.0 (GraphPad Software, San Diego, CA, USA).

Differences were considered statistically significant at *p* < 0.05.

## 3. Results and Discussion

### 3.1. Solubility of Dimethyl Fumarate in Geraniol

HPLC-based solubility studies demonstrated that DMF exhibits a higher solubility in GER than in water at room temperature (25 ± 1 °C), with 27.5 ± 0.97 mg of DMF dissolving in 1 mL of the oil phase. This value is approximately twenty times greater than its previously reported aqueous solubility under identical experimental conditions (about 1.4 mg/mL) [28], indicating an affinity of DMF for the lipophilic environment provided by GER. These results support the use of GER as a suitable solubilizing component for the development of DMF-loaded NEs.

### 3.2. Thermal Analysis

Thermogravimetric analysis (TGA) was performed to study the influence of the oil phase on DMF thermal stability.

**Figure 1:**
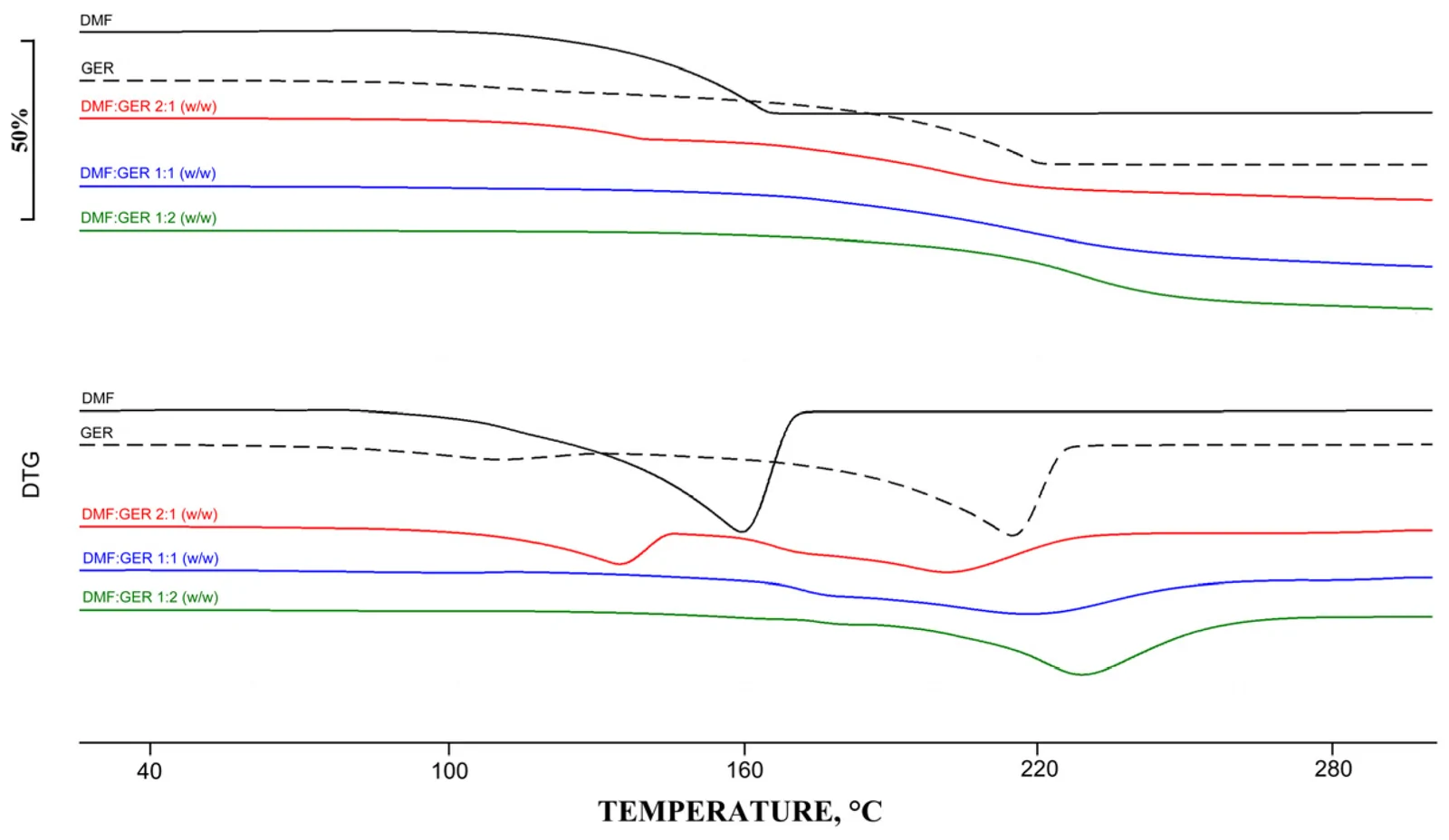
TGA curves (**above**) and first derivatives DTG (**below**) of DMF (black) and GER (black dotted) as such and of their physical mixtures at weight ratios of 2:1 (red), 1:1 (blue) and 1:2 (green), respectively.

In Figure 1, the TGA curves and the respective first derivatives (DTG) of DMF, GER, and their physical mixtures (PMs) at different DMF:GER weight ratios (2:1, 1:1, and 1:2, respectively) were compared.

The TGA curve recorded on pure DMF showed a single mass loss at about 125 °C, associated with the sublimation of the anhydrous sample. The PM with a 2:1 (w/w) DMF:GER ratio exhibited two different mass losses, the first of which was 25% and occurred between 57 and 143 °C, and could be attributed to the sublimation of excess DMF that was not completely stabilized by the oil phase, followed by a second mass loss of about 70%, in the range between 143 and 312 °C, corresponding to the decomposition of the remaining PM.

In contrast, the PM at the ratio 1:1 (w/w) exhibited only a mass loss of 90% starting at around 158 °C, and therefore higher than the sublimation temperature of pure DMF. This shift is consistent with a stabilizing effect exerted by the oil phase on DMF.

Also, the TGA of the PM at the ratio 1:2 (w/w) showed a shift in the DMF decomposition onset temperature to a higher value (T_onset_ = 200 °C), supporting the hypothesis that the oil phase exerts a stabilizing effect, directly proportional to its concentration in the formulation.

As already highlighted in our previous study on NEs with DMF and carvacrol [38], in all three samples the amount of DMF exceeds the solubility determined at 25 °C, although an increase in solubility may occur with the temperature increase during the experiment. The results therefore support the conclusion that DMF exhibits enhanced thermal stability at elevated temperatures when solubilized in the oil phase as compared to the pure drug.

**Figure 2:**
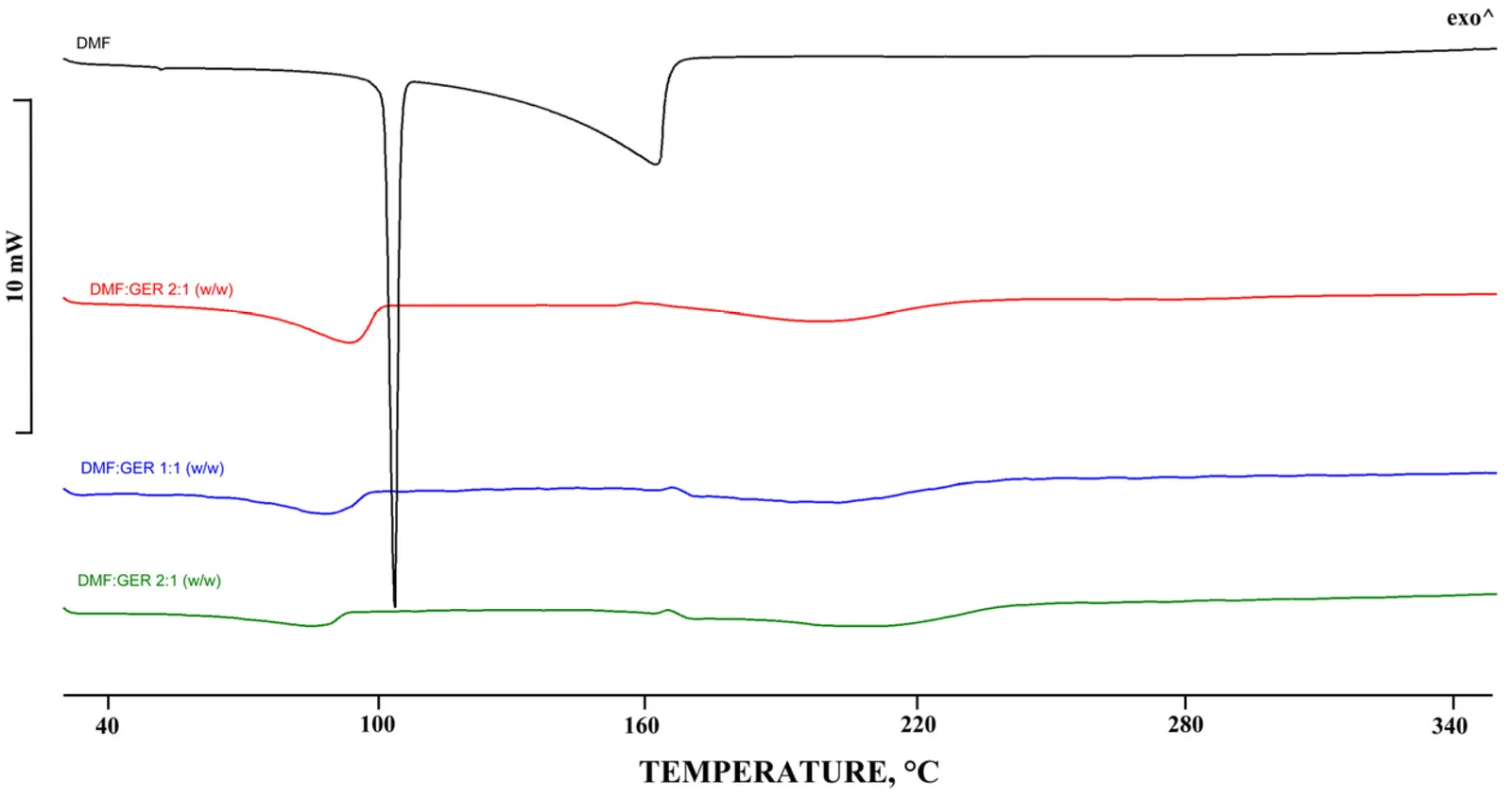
DSC curves of DMF (black) and its PMs with GER at ratios of 2:1 (w/w) (red), 1:1 (w/w) (blue) and 1:2 (w/w) (green), respectively.

DSC analysis was performed on DMF and on the same PMs. DMF showed a typical thermal profile of an anhydrous crystalline compound, with a sharp endothermic peak at 103.2 ± 0.7 °C, corresponding to its melting point (T_onset_ = 100.5 ± 0.5 °C; ∆H_fus_ = 208 ± 2 J/g). All DSC curves of the PMs displayed similar thermal profiles with a broadening and a shift to lower temperatures of the DMF melting peak as the amount of GER increased, consistent with its behavior as an ‘impurity’ within the system. Nevertheless, the normalized enthalpy of DMF remained nearly constant across the mixtures (∆H_fus_ = 200 ± 4 J/g) and comparable to that of the pure DMF, confirming the homogeneity of the mixtures (Figure 2).

### 3.3. Preparation of the Nanoemulsions

NEs can be prepared using either high-energy or low-energy emulsification methods, each offering distinct advantages [67]. High-energy methods, including ultrasonication and homogenization, require mechanical forces to break down larger droplets and achieve smaller particle sizes [33]. Ultrasonication uses high-frequency sound waves (≥20 kHz) to create cavitation, forming and collapsing microdroplets that generate nano-sized emulsions [68]. This method effectively reduces droplet size while preserving NE integrity [69]. Similarly, high-pressure homogenization applies intense mechanical forces to break down droplets, aiding in homogenization, emulsification, and suspension production [70]. While effective, homogenization faces challenges in automation and scalability. By optimizing these methods, stable and efficient NEs can be developed for drug delivery applications, including the intranasal administration of therapeutics targeting the CNS.

In contrast, low-energy methods, such as phase inversion and spontaneous emulsification, leverage the physicochemical properties of components to form emulsions without external energy input [71]. Considering the very poor stability of DMF [13,27], we preferred these methods over the high-energy ones. Phase inversion emulsification can be induced by either composition changes (PIC) or temperature shifts (PIT), leading to applications in the pharmaceutical, cosmetic, and food industries [72,73]. Spontaneous emulsification relies on surfactant diffusion between phases and has been used to develop delivery systems for bioactive compounds, achieving particle sizes below 200 nm [74,75]. While these NEs can be stable at room temperature, co-surfactants may enhance their thermal stability [76]. Given its simplicity and cost-effectiveness, this method holds promise for pharmaceutical and biomedical applications, including nasal drug delivery [77].

The NEs were prepared using CSOA as a surfactant, taking into account that chitosan-coated formulations further enhance drug stability, controlled release, and bioavailability across various routes. It is well known that chitosan-coated nanoparticles exhibit high encapsulation efficiency and positive zeta potential, which allow them to improve their interaction with biological membranes [78], making them promising for drug delivery applications [79].

#### 3.3.1. Phase Inversion Emulsification Method

The first method used for the preparation of NEs was the phase inversion method combined with an Ultra-Turrax homogenizer (PI). The droplet size of the internal phase was evaluated through a unimodal analysis combined with a numerical size distribution, both obtained from PCS.

**Figure 3:**
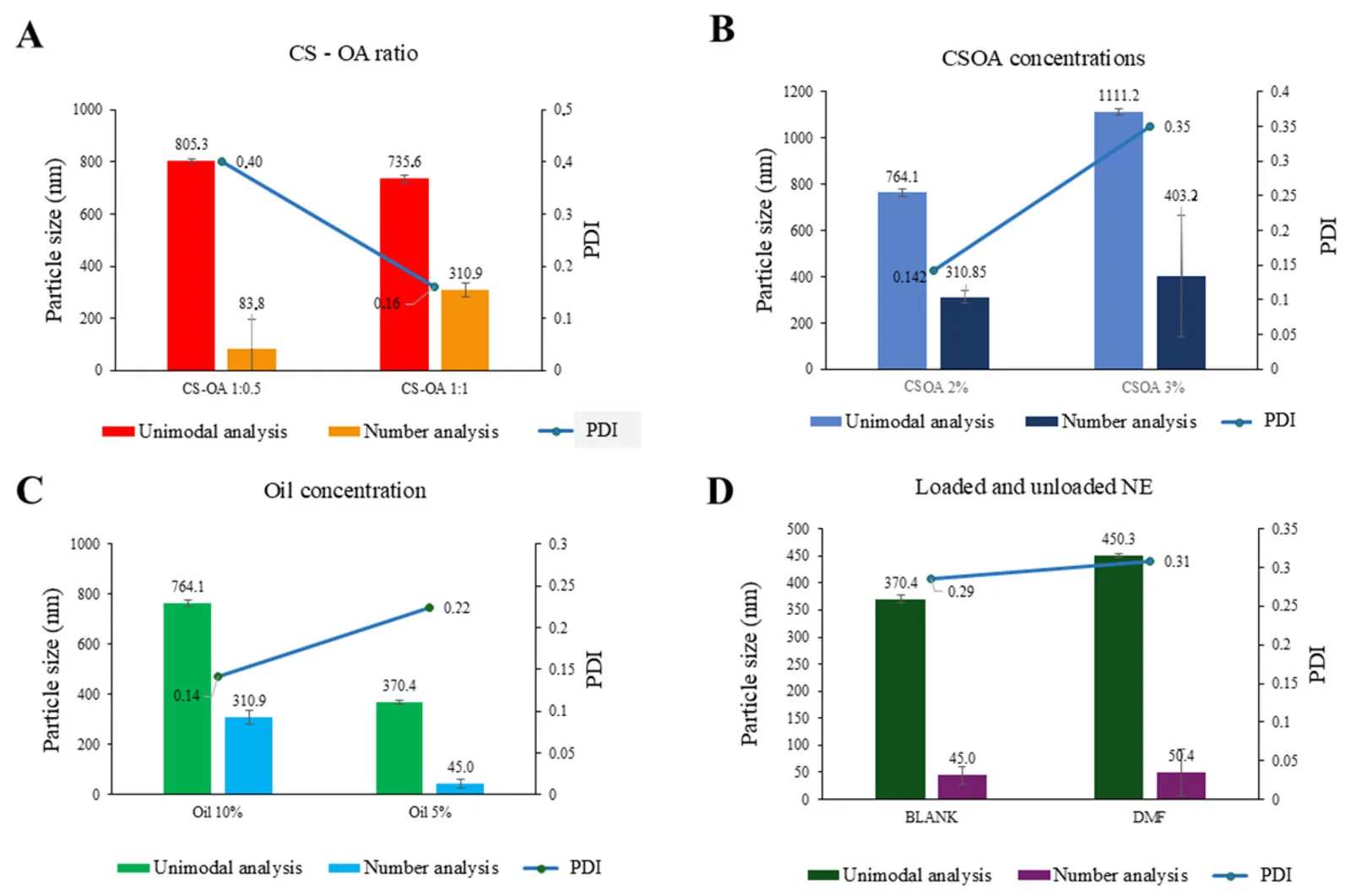
Effects of formulation parameters on the mean droplet size and polydispersity index (PDI) of NEs prepared by the phase inversion method. The dimensional properties were evaluated as a function of (**A**) the CSOA ratio (1:0.5 vs. 1:1), (**B**) the 1:1 CSOA concentration (2% vs. 3%), (**C**) the oil phase content (GER 10% vs. 5%), and (**D**) the presence or absence of 2% DMF with respect to the oil phase. Mean size values are expressed as given intensity (unimodal analysis) and number (numerical analysis) (mean ± SD, *n* = 3).

In Figure 3, the results are given as mean droplet size and PDI. The effect of two different chitosan (CS) and oleic acid (OA) molar ratios (1:0.5 and 1:1) on particle size was first assessed by PCS. As illustrated in Figure 3A, in the case of size distribution in intensity, the mean diameter of the internal phase droplets of the NE prepared with CSOA in a 1:1 ratio was slightly smaller (736 ± 15 nm) compared to that of the formulation with a CSOA 1:0.5 ratio (805 ± 7 nm). On the other hand, the PDI values were significantly lower in the formulation with a CSOA 1:1 ratio, indicating a narrower size distribution and improved homogeneity (Student’s *t*-test, *p* < 0.05).

The discrepancy observed between the mean diameter values obtained by intensity and by number is therefore probably attributable to the higher polydispersity of the sample with CSOA 1:0.5 compared to the 1:1 formulation. Based on these results, the CSOA molar ratio of 1:1 was selected for further study, as it resulted in a more uniform and homogeneous NE, in terms of droplet size distribution.

Subsequent studies instead focused on comparing NE formulations with two different concentrations of 1:1 CSOA, namely 2% and 3% (w/v) (Figure 3B). Increasing the CSOA concentration to 3% led to a significant increase (nearly twofold) in the mean diameter of the internal phase droplets, with a particle size of 1111 ± 14 nm compared to 764 ± 15 nm for the 2% CSOA formulation, in terms of intensity (unimodal analysis). Similarly, the size distribution by number revealed larger droplets for the 3% CSOA formulation (403 ± 261 nm) than those in the 2% formulation (311 ± 27 nm), although the high variability observed in the 3% samples made this difference statistically insignificant. This behavior may be attributed to the aggregation of the excess surfactant molecules in the continuous phase, which reduces the effective amount of surfactant available to stabilize the oil–water interface, leading to droplet coalescence and size increase [80]. In addition, the NE prepared with the 2% CSOA displayed the lowest PDI, thereby justifying the selection of this concentration for further formulation.

Figure 3C, by contrast, showed the effect of reducing the oil phase concentration (GER) from 10% (500 mg) to 5% (250 mg). A significant decrease in the NE mean droplet size was observed at the lower concentration of oil, as evidenced by both unimodal and numerical size analyses (Student’s *t*-test, *p* < 0.05). Although the PDI of the 5% GER formulation was slightly higher, it remained within the acceptable range, with an average value of 0.22. Based on these results, the 5% GER concentration was selected for further studies. These results agree with the literature [81,82] indicating that an increase in oil content in NEs increases the viscosity of the oil phase, thereby reducing its ability to disperse effectively within the aqueous phase.

The NE, based on a CSOA 1:1 ratio with CSOA at 2% and GER at 5%, was therefore loaded with DMF at 2% relative to the oil phase. The influence of this loading on the droplet size of the dispersed phase was then evaluated. Accordingly, dimensional analyses were performed on both blank and 2% DMF-loaded NEs (Figure 3D). The results showed a slight increase in mean droplet size when the oil phase of the formulation was loaded with DMF. The PDI values remained comparable between the two formulations, indicating preserved droplet uniformity. Nonetheless, the differences in particle size and PDI were statistically significant (ANOVA, Fisher’s post hoc test, *p* < 0.05), indicating that DMF loading influences the dimensional characteristics of the NE system.

The formulation identified as optimal using the PI method was subsequently subjected to a stability study to evaluate its physicochemical robustness over time.

##### Dimensional Stability Studies

**Figure 4:**
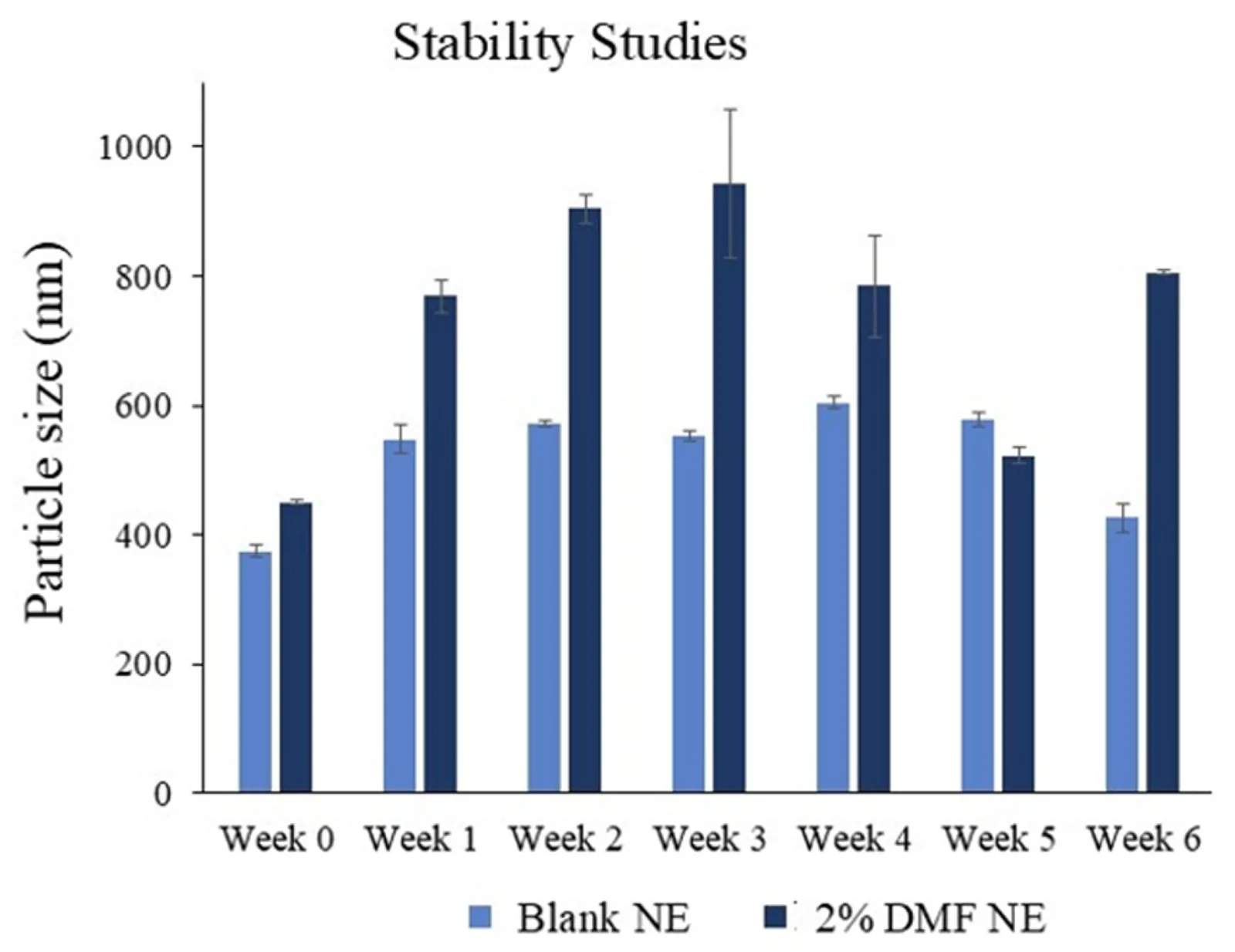
Comparison of the mean droplet size of blank NEs and NEs loaded with 2% DMF in oil phase obtained by PI and formulated with 2% CSOA (1:1) and 5% GER. Stability was monitored over 6 weeks storage period at 4 ± 1 °C (mean ± SD, *n* = 3).

Dimensional stability analyses were performed on both loaded and unloaded NEs, stored for six weeks at 4 ± 1 °C. As shown in Figure 4, the DMF-free NEs (i.e., blank NE) exhibited an increase in droplet size during the first week of storage (<200 nm), after which the mean diameter remained substantially unchanged throughout the remainder of the study. In contrast, the DMF-loaded NEs showed a marked increase in droplet size, from approximately 450 nm at preparation to 943 nm after four weeks of storage.

#### 3.3.2. Spontaneous Emulsification Method

**Figure 5:**
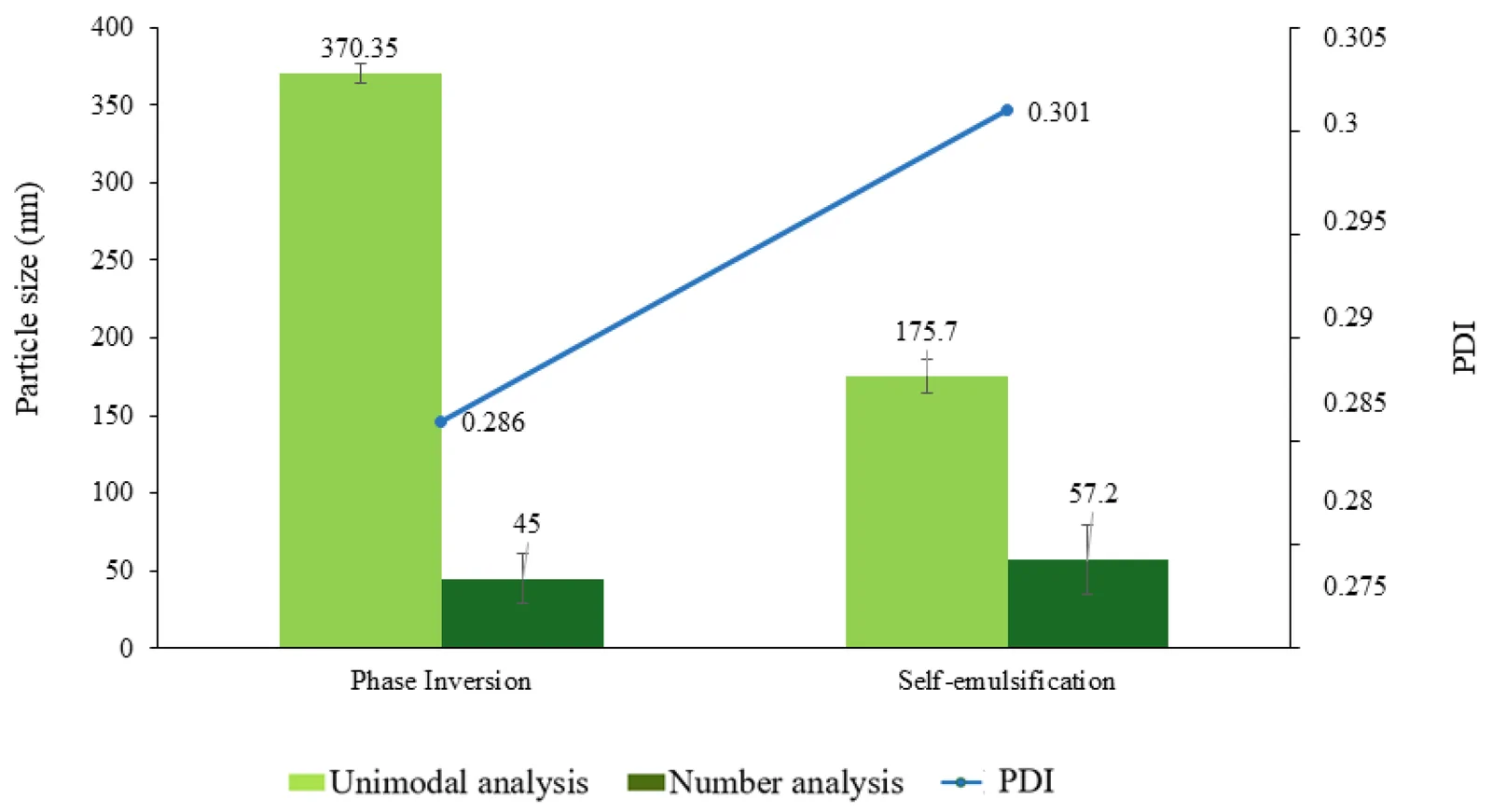
Comparison of the average droplet size and PDI of blank NEs prepared by PI (left) and SE (right) methods and containing 2% 1:1 CSOA and 5% GER. Mean size values are expressed as given intensity (unimodal analysis) and number (numerical analysis) (mean ± SD, *n* = 3).

To improve the stability of the formulation, a second preparation method was investigated, specifically spontaneous emulsification (SE). The NEs obtained with this method had a translucent appearance and a bluish color, according to the Tyndall effect characteristic of nano-dispersed systems. As shown in Figure 5, the droplet size obtained for the blank NE prepared by SE was smaller than that obtained using the PI method; while the PDI remained around 0.3, indicating good size homogeneity.

A statistically significant difference was observed in the intensity and PDI values between NEs prepared by the two methods (Student’s *t*-test, *p* < 0.05). Based on these results, SE-prepared formulations were selected for further investigations.

Considering the very poor stability of DMF [13,27,28], some advantages can be envisaged for this method over the high-energy one.

**Figure 6:**
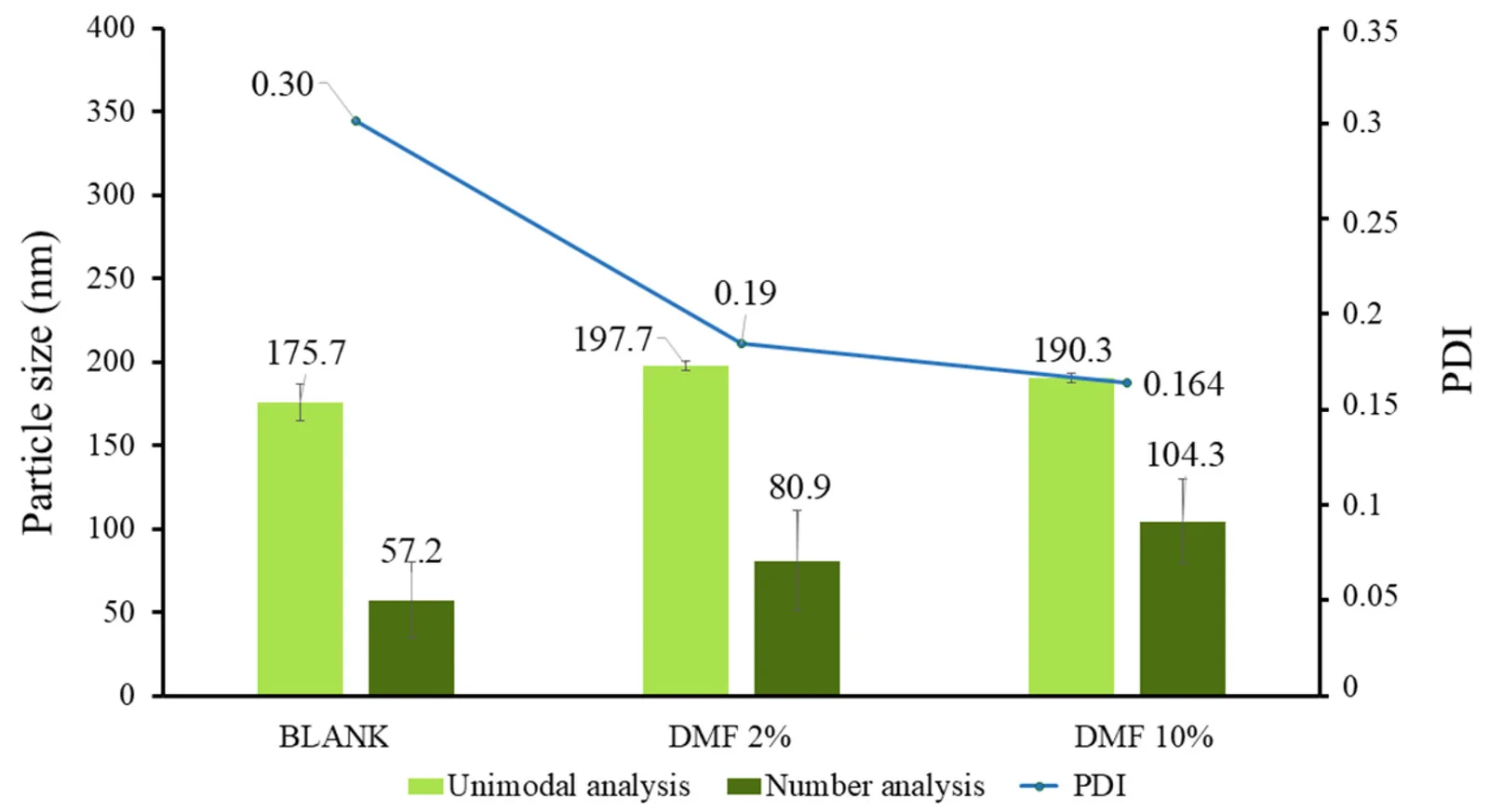
Comparison of the average droplet size and PDI of blank NEs and DMF-loaded NE prepared by the SE method with both 2 and 10% DMF with respect to the oil phase. Mean size values are expressed as given intensity (unimodal analysis) and number (numerical analysis) (mean ± SD, *n* = 3). Statistically significant differences were observed only for unimodal analysis between blank vs. DMF 2% and blank vs. DMF 10% (one-way ANOVA and Fisher’s post hoc test, *p* < 0.05).

Figure 6 shows a comparison between unloaded and DMF-loaded NEs prepared via SE, at 2% (v/v) of DMF in the oil phase and at a higher concentration of up to 10% (v/v). Both loaded NEs exhibited a slight increase in droplet size (observed in both unimodal and numerical analyses) compared to the corresponding blank NEs. The lower PDI of the DMF-loaded NEs indicated a more monodisperse and homogeneous droplet population. Statistical analysis evidenced that the differences in unimodal analysis between unloaded and loaded formulations, although quite small, were statistically significant, while no statistical differences were found in the case of number analysis (one-way ANOVA followed by Fisher’s LSD post hoc test, *p* < 0.05).

Considering the good dimensional results, the subsequent characterization was performed on the NE loaded with 10% DMF. This formulation corresponded to a theoretical DMF concentration of 0.24 mg/mL and a GER concentration of 2.4 mg/mL. The quantification of DMF and GER in this formulation after preparation was verified by HPLC and resulted in a recovery of about 74 ± 8% and 82 ± 3% for DMF and GER, respectively.

### 3.4. Fourier Transform Infrared Analysis

**Figure 7:**
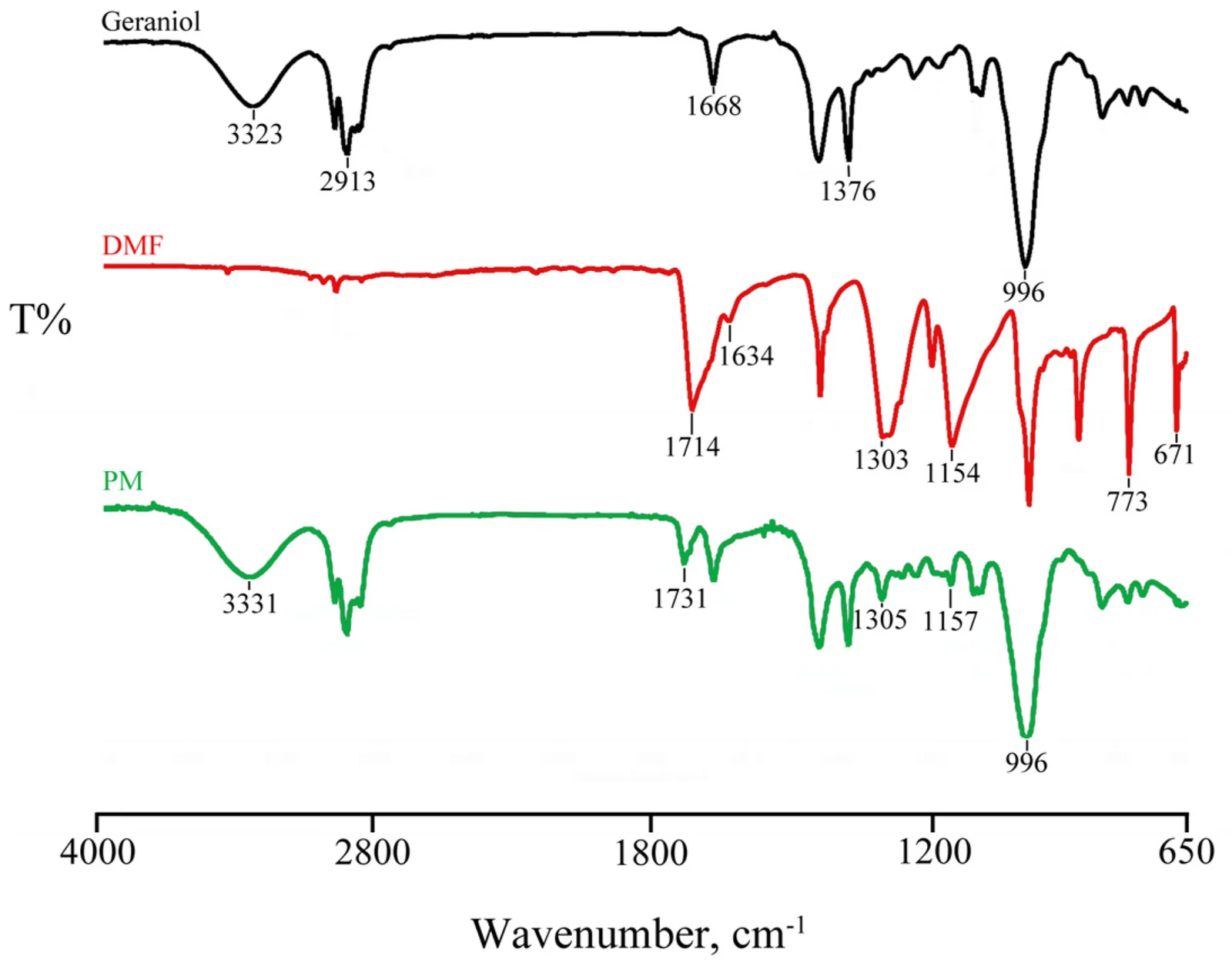
FT-IR spectra of GER (black), DMF (red) and their physical mixtures containing 10% of DMF (green).

The interactions occurring between the substances within the NEs were further characterized by FT-IR. First, to identify possible interactions between the two compounds, the FT-IR spectra of pure GER, pure DMF, and their PMs containing 10% (w/w) DMF in the oil phase were compared (Figure 7).

According to the literature, the FT-IR spectrum of GER shows a broad band at 3323 cm^−1^ due to the stretching vibration of the terminal -OH group. Additional bands at 2913 and 1376 cm^−1^ are attributed to -CH_2_ stretching vibrations, while the band at 1668 cm^−1^ corresponds to the conjugated double bond C=C. The band at 996 cm^−1^ is associated with the out-of-plane bending vibration of the -CH group [83,84].

In the DMF spectrum, characteristic bands are observed for the carbonyl C=O stretch at 1714 cm^−1^, the C=C stretch at 1634 cm^−1^, and C-O ester stretching vibrations at 1154 and 1303 cm^−1^. Additional bands at 773 and 671 cm^−1^ correspond to -CH bending vibrations.

Some of these bands shift to higher wavenumbers in the spectrum of the PMs, suggesting the occurrence of molecular interactions between the two components. Specifically, the carbonyl stretching band of DMF at 1714 cm^−1^ shifts to 1731 cm^−1^ in formulations containing 10% DMF. Similarly, the bands at 1303 and 1154 cm^−1^ shift to 1305 and 1157 cm^−1^, respectively. These spectral changes indicate alterations in the chemical environment of the two components, consistent with interactions between DMF and GER.

**Figure 8:**
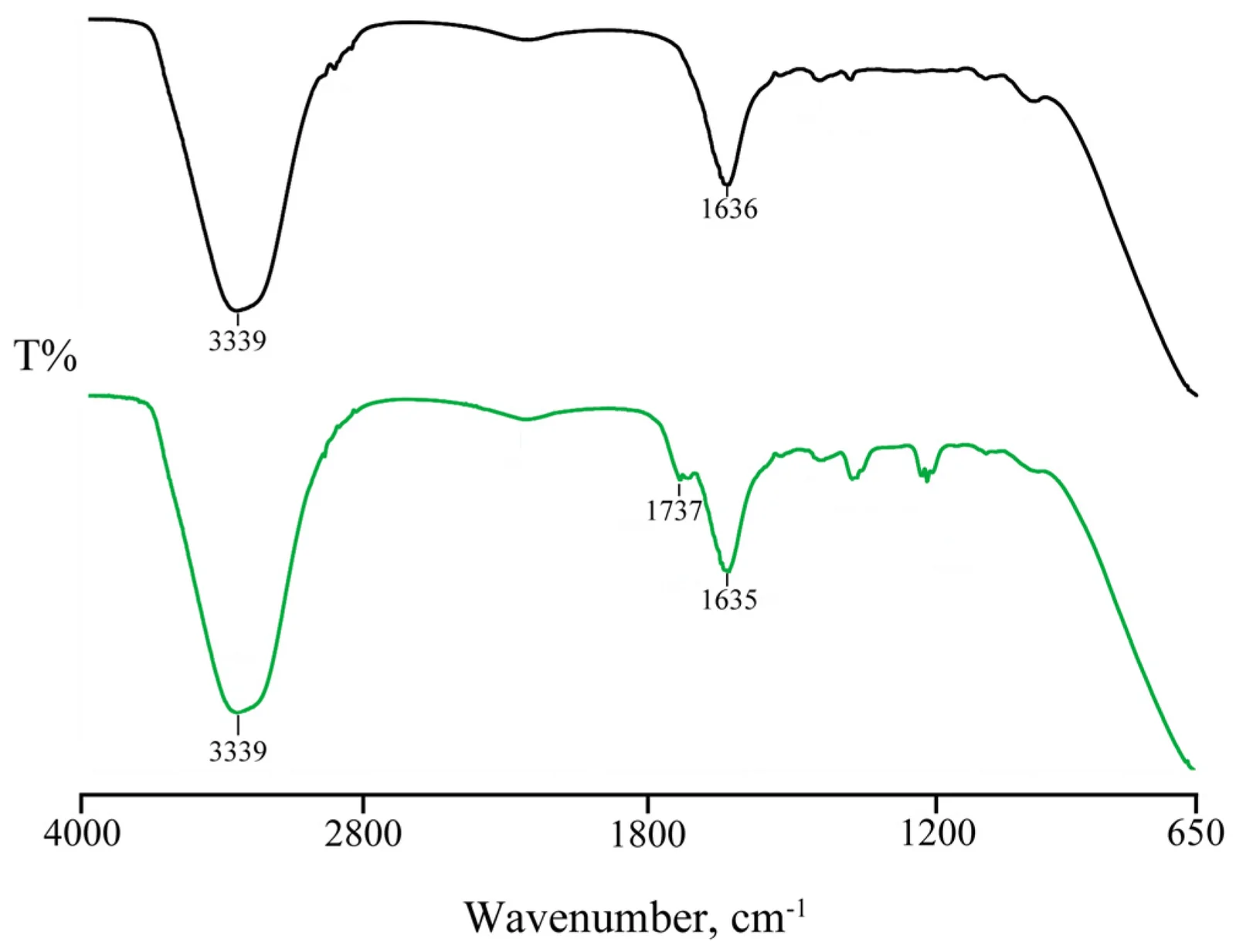
FT-IR spectra of blank NE (black) and 10% DMF-loaded NE (green), both containing 2% CS-OA 1:1 and 5% (w/v) GER.

FT-IR spectra of the blank NEs and NEs containing 10% DMF, are presented in Figure 8. In both spectra, the predominant absorption region appeared between 3600 and 3200 cm^−1^, with a peak at around 3330 cm^−1^, corresponding to the stretching vibration of the water O-H groups and reflecting the high hydration level of the O/W NE. This was followed by a band at 1636 cm^−1^ attributable to the bending vibration of O-H groups, indicating inter- and intramolecular hydrogen bonds occurring during NE formation.

In the DMF-loaded NE spectrum, an additional band at 1737 cm^−1^, corresponding to the characteristic carbonyl C=O stretching vibration, was observed, confirming the incorporation of the active ingredient into the formulation.

### 3.5. Dimensional Stability Studies

The dimensional stability of the DMF-loaded NE was evaluated over three months of storage under refrigerated conditions (4 ± 1 °C). During this period, the formulations were monitored for changes in droplet size distribution. The particle size differences among NEs (blank vs. DMF-loaded) prepared by the SE method were statistically significant (*p* < 0.05, one-way ANOVA).

In general, formulations characterized by smaller droplet sizes exhibited greater stability during storage, which is in agreement with previous studies [81]. Moreover, all formulations remained stable during the first four weeks, and droplet sizes did not exceed 400 nm.

**Figure 9:**
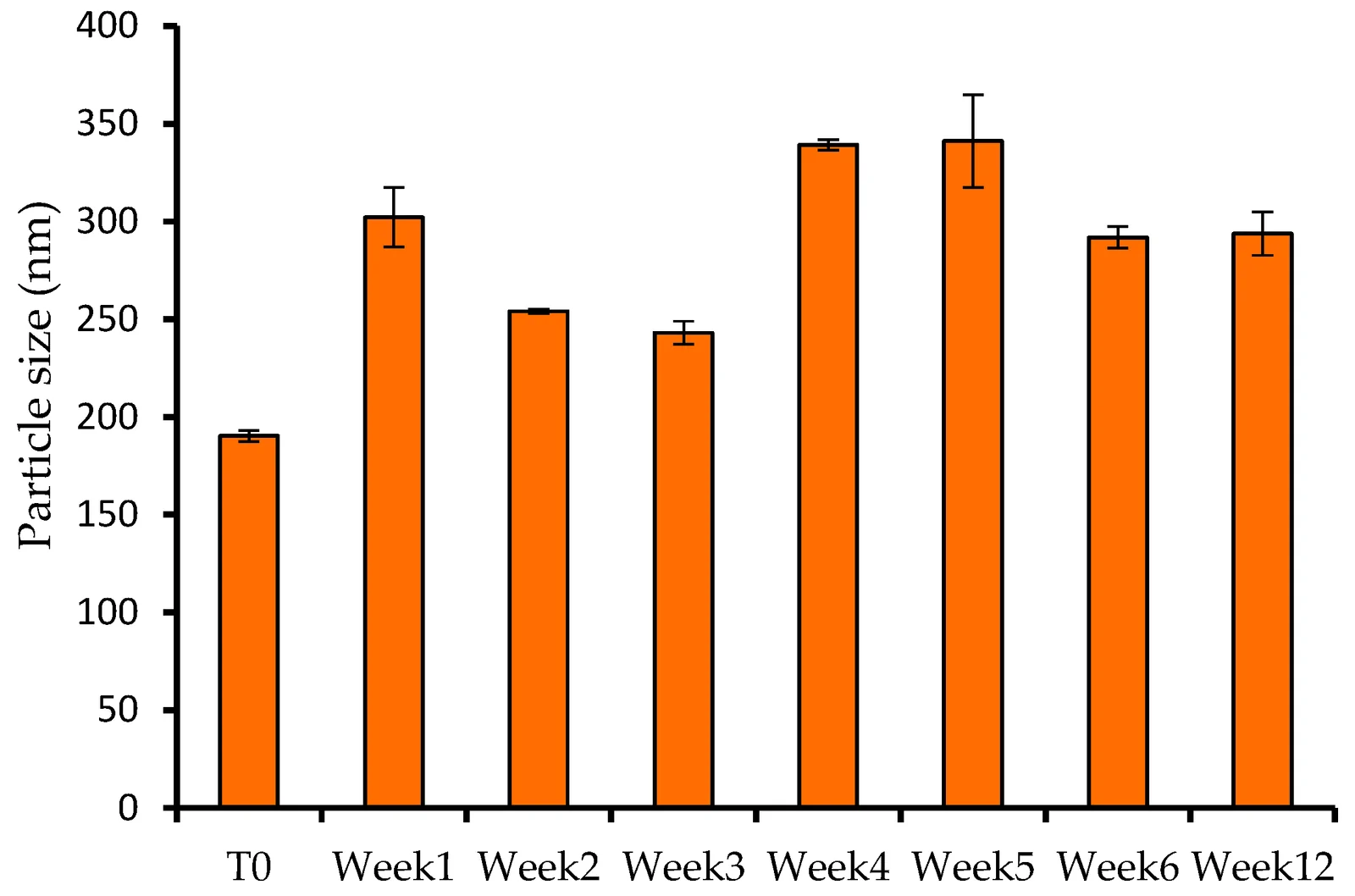
Droplet size of 10% DMF-loaded NEs prepared by SE method (mean ± SD, *n* = 3).

Among the three formulations, the NE containing 0.24 mg/mL DMF (corresponding to 10% DMF in the oil phase) remained the most stable during the first four weeks, exhibiting a smaller droplet size (<350 nm) compared to the other formulations as reported in Figure 9.

**Figure 10:**
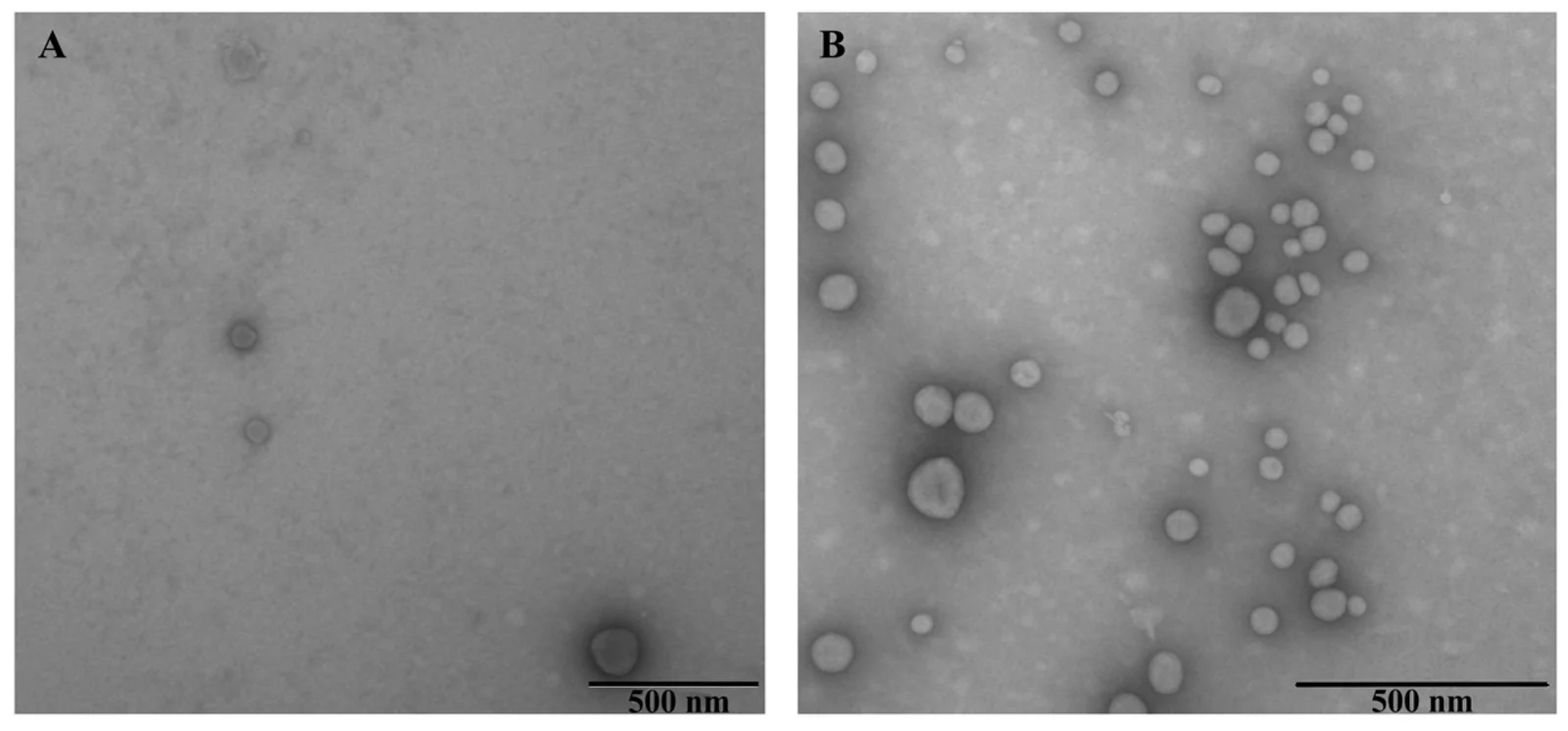
TEM images of blank NE (**A**) and 10% DMF-loaded (**B**) NE, both stained with uranyl acetate.

TEM analysis was performed to characterize the unloaded and loaded NEs in terms of size and their morphology. The NE images obtained confirmed the droplet size (~170 nm) and the spherical shape of the internal phase droplets for both blank and loaded NEs (Figure 10).

Specifically, the blank NEs appeared as spherical globules with definite dark boundaries and a lighter inner region (A), whereas the DMF-loaded NEs exhibited spherical droplets with a dark inner core. Overall, the droplet size observed by TEM for both types of NEs was found to be comparable to that obtained by PCS analysis, confirming the validity of the size distribution data.

### 3.6. Zeta Potential and pH of Nanoemulsion

Zeta potential measurements were carried out to assess the long-term stability of NE formulations and to understand the state of the surface morphology. The blank NE exhibited a zeta potential of 20 ± 8 mV, while the DMF-loaded NEs exhibited a comparable value of 22 ± 10 mV. The positive zeta potential is consistent with the presence of CSOA on the particle surface. This is indicative of a good physical stability of the dispersed system by preventing droplet aggregation through electrostatic repulsion. Moreover, the positive surface charge is relevant for interaction with mucin and cell membranes, potentially enhancing mucoadhesion and improving bioavailability, as indicated by the literature [85,86].

Finally, the pH of this NE formulation was assessed, showing a value of 5.5 ± 0.5, which is compatible with the physiological pH range of the nasal mucosa (4.5–6.5) [87].

### 3.7. In Vitro Release Studies

The in vitro dialyzing membrane model was used to evaluate the release kinetics of DMF and GER from the NEs. Analysis of the release profile obtained revealed a rapid initial release phase within the first 120 min, during which 85 ± 4% of the total encapsulated DMF was released in good accordance with the previous results obtained with a NE formulation combining DMF and carvacrol [88]. The cumulative release reached 87 ± 4% at 210 min. When compared with free DMF, which under the same experimental conditions showed a cumulative release of 85 ± 2% after 60 min in a previous study, the NE formulation exhibited a slightly slower release profile [88]. However, the release remains compatible with the nasal application, which requires relatively fast release to optimize absorption during the short contact time of the formulation on the nasal mucosa.

Analogous considerations can be made for GER, which showed a maximum cumulative release of 84 ± 5% after 210 min.

Overall, these results confirm that the release behavior is suitable for nasal administration, considering that the nasal mucociliary clearance occurs in about 10–20 min, but this time can be prolonged due to the chitosan mucoadhesion effect. Nose-to-brain absorption is based to a relevant extent on the possible extraneuronal pathway to the CNS, by which the drugs can be transported along olfactory neurons in a short time (<30 min). This pathway should be active also for the cargo released in the nasal olfactory epithelium without the nanocarrier endocytosis. However, in the case of endocytosis, the transport of the carrier would be much slower, taking several hours or days [89,90]. In the present case, considering that the release is completed in a few hours, it is likely that the passage to the CNS involves the NE cargoes, after their release.

### 3.8. ROS-Scavenging Activity

**Figure 11:**
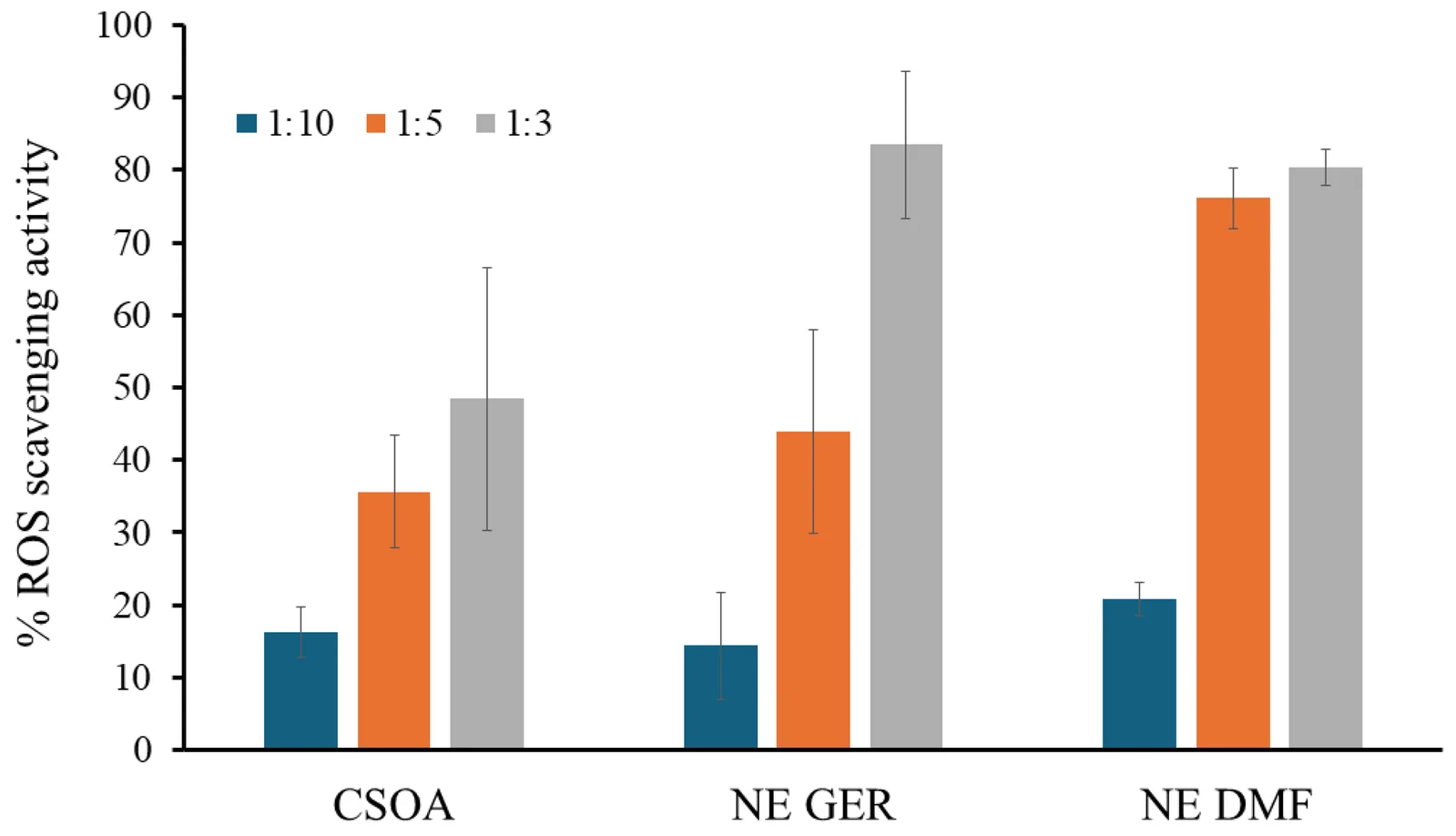
ROS-scavenging activity % of CSOA, blank NE (NE GER) and DMF-loaded NE (NE DMF). Statistically significant differences among groups with equivalent dilution ratio were only found for: CSOA 1:5 vs. NE DMF 1:5; NE GER 1:5 vs. NE DMF 1:5; CSOA 1:3 vs. NE GER 1:3; and CSOA 1:3 vs. NE DMF 1:3 (one-way ANOVA, Fisher’s least significant difference (LSD) procedure, *p* < 0.05).

The results of the antioxidant activity of DMF-loaded NE (NE DMF), blank NE (NE GER) and CSOA are shown in Figure 11. In general, the samples showed a statistically significant dose-dependent ROS-scavenging activity (one-way ANOVA, Fisher’s least significant difference (LSD) procedure, *p* < 0.05). For all the samples, statistically significant increase was found between the 1:10 and 1:5 dilutions (*p* < 0.05). Notably, the CSOA and DMF-loaded NE reached their maximum activity already at a 1:5 dilution, as the difference between the 1:5 and 1:3 dilutions is not statistically significant for these formulations. For the blank NE GER, a comparable effect with NE DMF was found only with the highest concentration (1:3 dilution). This difference is probably due to the combined presence of DMF and GER in the loaded NEs. The essential oil is an acyclic isoprenoid monoterpene, which, as demonstrated in the literature, already exhibits intrinsic antioxidant properties, possibly due to its hydroxyl group responsible for ROS deactivation [91,92]. Furthermore, DMF itself is known to exert a Nrf2-mediated antioxidant effect, especially in neuroinflammation [93].

### 3.9. Cytotoxicity Studies

**Figure 12:**
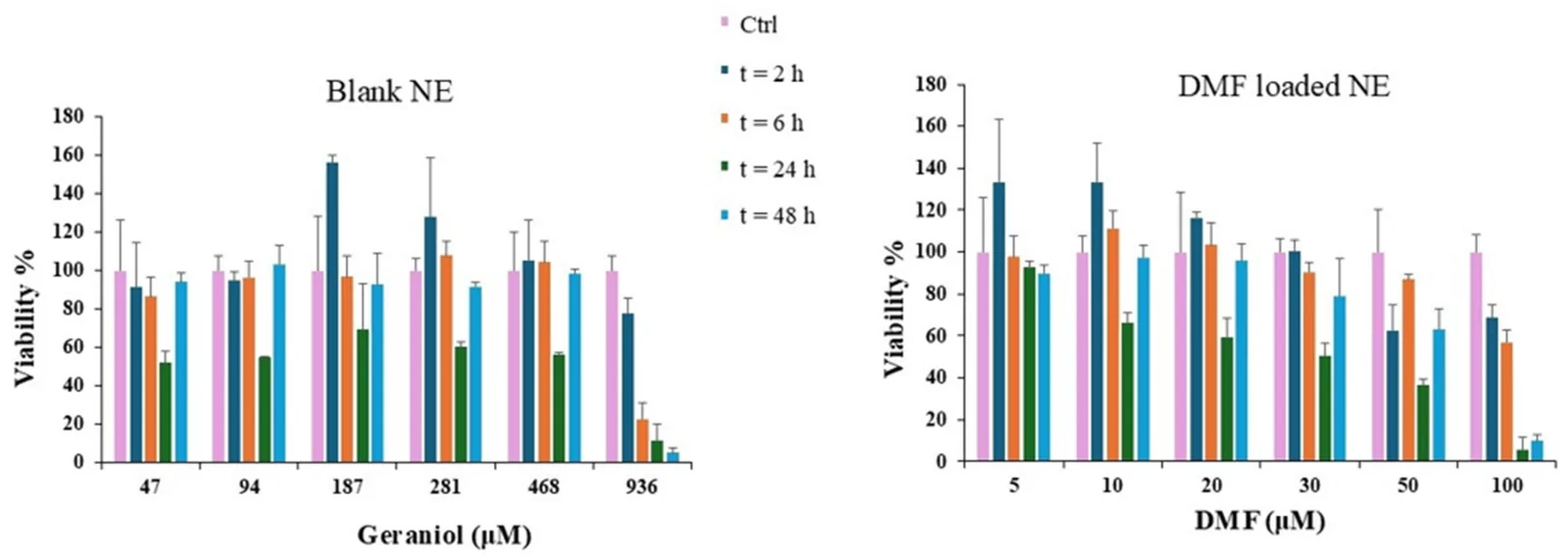
Viability % of RPMI 2650 cells following incubation with increasing concentrations of blank NE and DMF-loaded NE at different concentrations (mean ± SD, *n* = 3).

Cellular metabolic activity was evaluated using the MTT assay on RPMI 2650 cell line treated with DMF-loaded NE (obtained with the SE method), blank NE, and free drug at different incubation times (2, 6, 24, and 48 h) and DMF concentrations (5, 10, 20, 30, 50, 100 μM). The results are shown in Figure 12. Overall, the data demonstrated that the cytotoxicity profile was concentration- and time-dependent. Good short-term cytocompatibility was observed at concentrations up to 50 μM, whereas a marked loss of cell viability occurred at the highest concentration tested (100 μM), particularly after 24 h of exposure.

Although a general dose-dependent decrease in cellular metabolic activity was observed, RPMI 2650 cells exhibited higher metabolic activity after 48 h of incubation with both blank and DMF-loaded NEs compared with the corresponding values measured after 24 h. This observation suggests that prolonged exposure to the formulations did not impair cellular metabolic activity and may even promote metabolic activity in RPMI 2650 cells under the tested conditions.

The lowest viability value (10 ± 3%) was observed after 24 h for the formulation with the highest DMF concentration (100 μM).

The results demonstrated high cytocompatibility of the loaded NE, with cell viability values above 87 ± 3% up to a 50 μM after 6 h of incubation. Based on these results, the samples containing 50 μM DMF were selected for the following in vitro permeability assay.

### 3.10. Permeability Studies

To evaluate the ability of NEs prepared with the two different methods to permeate the nasal epithelium, permeability studies were conducted using RPMI 2650 cells cultured at the air–liquid interface, a model that is, according to the literature, widely recognized as an in vitro analog of the human nasal epithelial barrier [56].

**Figure 13:**
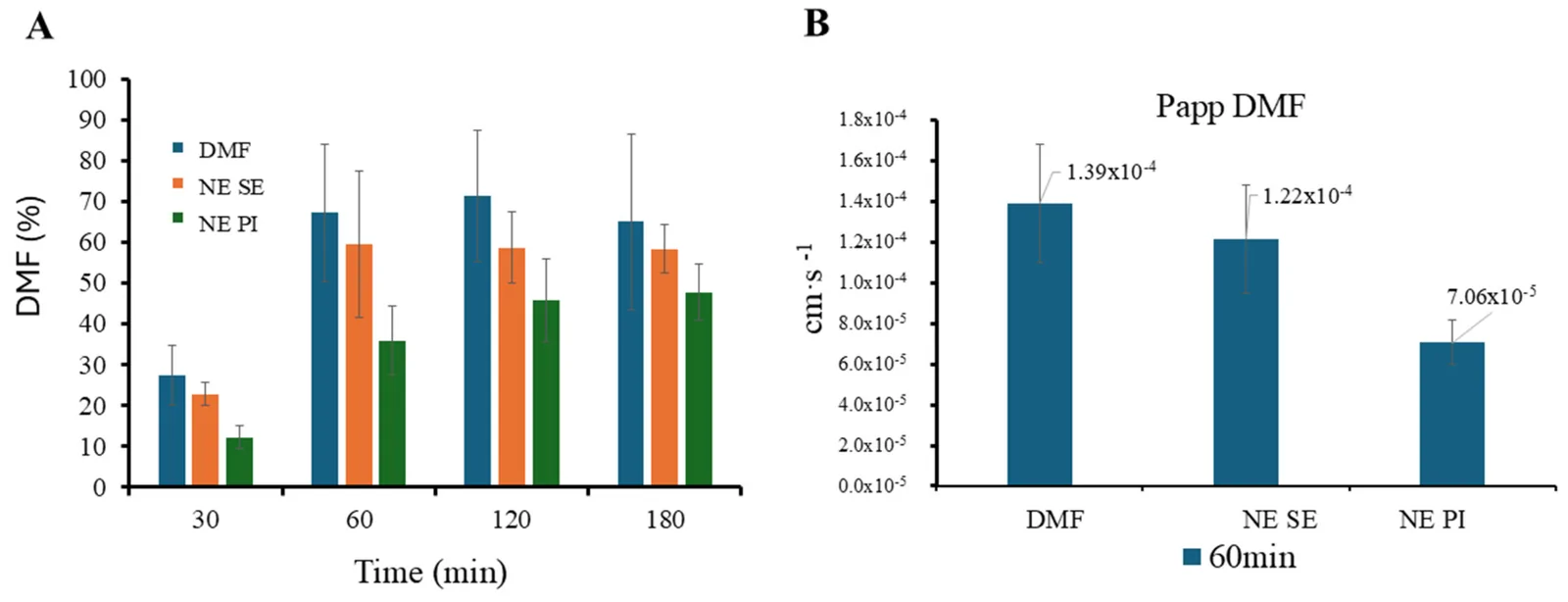
(**A**) Permeability of DMF (blue), DMF-loaded NE prepared by SE method (orange) and by PI (green) on RPMI 2650 cell monolayer at air–liquid interface. Statistically significant differences for corresponding times: DMF vs. NE SE 120 min, DMF vs. NE PI all times, NE SE vs. NE PI 30 and 60 min (one-way ANOVA, Fisher post hoc, *p* < 0.05). (**B**) Apparent permeability coefficient of permeated DMF after 60 min (mean ± SD, *n* = 4). Statistically significant differences: DMF vs. NE PI and NE SE vs. NE PI (one-way ANOVA, Fisher post hoc, *p* < 0.05).

The DMF solubilized in a methanol:culture medium mixture (30:70 *v*/*v*) and then diluted in culture medium to obtain a 50 μM concentration was tested as a control. The permeability data are shown in Figure 13A. After 60 min, free DMF exhibited the highest permeation, reaching nearly 70%, while the NE produced by the SE method (NE SE) and that produced by PI (NE PI) reached 59% and 35%, respectively. This trend was maintained up to 120 and 180 min, with free DMF permeability around 70%, NE SE remaining stable at 59%, and NE PI progressively increasing to 45%.

Free DMF exhibited the greatest variability (higher values of SD), particularly at 60 and 120 min, probably due to its low intrinsic stability, tendency to sublime, and poor solubility. 

Free DMF showed the highest apparent permeability under the experimental conditions, indicating that incorporation into the NE did not enhance DMF permeation relative to the unformulated drug. However, among the two NE formulations, NE-SE showed consistently greater permeability than NE-PI. Since the two formulations had the same qualitative composition but differed in their preparation method and resulting droplet characteristics, the higher permeability of NE-SE may be related, at least in part, to its smaller droplet size and narrower size distribution.

The calculated Papp values quantified these results and showed that free DMF solubilized in methanol demonstrated the highest permeability compared to the other two NE formulations (Figure 13B). Generally, a compound solubilized in an organic solvent, like methanol in this case, displays higher apparent permeability, due to the solvent’s ability to enhance solubility and decrease mass transfer resistance across the cell monolayer [94].

However, the difference between the percentage permeated for free DMF solubilized in methanol and DMF delivered by NE SE at 30 and 60 min was not statistically significant. It is important to underline that DMF’s poor solubility limits its bioavailability if not properly formulated.

Moreover, the results clearly highlighted that the droplet size of the dispersed phase plays a critical role in the delivery of the drug encapsulated in the GER phase. The smaller NE SE dimensions are a factor recognized as relevant for the improvement of drug absorption by the nose-to-brain route [86]. It can be envisaged that smaller dimensions correspond to a higher specific surface area of the droplets, larger contact area of the NE with the epithelium, and easier partition from the NE to the epithelium of both the oil and the drug loaded in the NE, resulting in faster absorption. After 3 h, however, the absorption percentage for the two NE samples became comparable.

**Figure 14:**
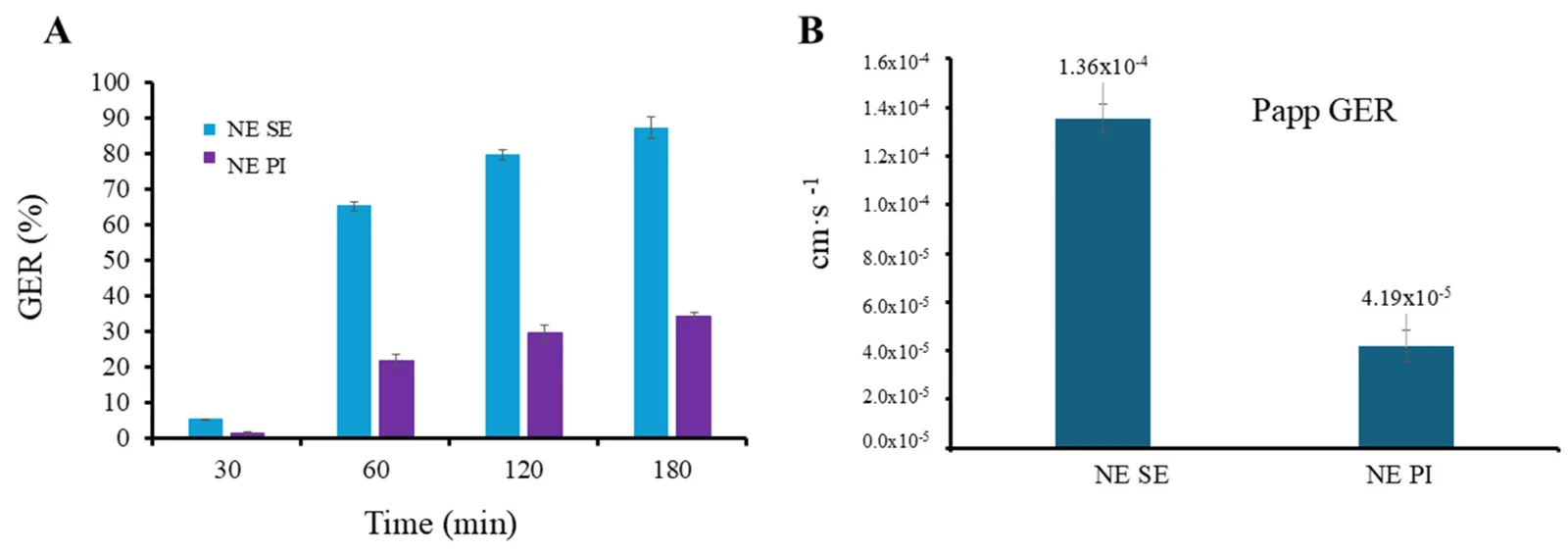
(**A**) Permeability of GER from NE prepared by SE method (sky blue) and by PI (purple) on RPMI 2650 cell monolayer. Statistically significant differences for corresponding times: NE SE vs. NE PI at 60, 120, and 180 min (one-way ANOVA, Fisher post hoc, *p* < 0.05). (**B**) Apparent permeability coefficient of permeated GER after 60 min (mean ± SD, *n* = 3). Statistically significant differences for corresponding times: NE SE vs. NE PI (Student’ *t*-test, *p* < 0.05).

A similar positive effect of smaller oil droplet dimensions was observed for GER absorption, as shown in Figure 14A. After one hour of incubation, GER from NE SE reached a maximum permeability of 65%, increasing to 79% and 87% at 120 and 180 min, respectively. In contrast, GER from NE PI reached only 34%, as a consequence of the larger droplet size of this formulation. Papp values calculated for GER at 60 min, and reported in Figure 14B, confirmed again the more marked difference between samples with different droplet sizes.

### 3.11. Formulation for In Vivo Study

As a proof of concept, a preliminary in vivo evaluation was performed in a rat model to evaluate the impact of the delivery system on DMF bioavailability in the CNS when administered by the nasal route together with GER.

To enable the in vivo evaluation, additional formulation studies were performed to increase the final DMF concentration in the NE based on the results obtained in the first part of the work. The target DMF concentrations were selected taking into account previous pharmacokinetic studies on intranasal DMF administration in rats, in which a dose of 1 mg/kg was employed [27]. The theoretical GER concentration was maintained constant at 5% (*v*/*v*), whereas the target DMF concentration was progressively increased from the formulation previously investigated (0.24 mg/mL; NE0.24) to 1.0 mg/mL (NE1) and 2.5 mg/mL (NE2.5).

**Figure 15:**
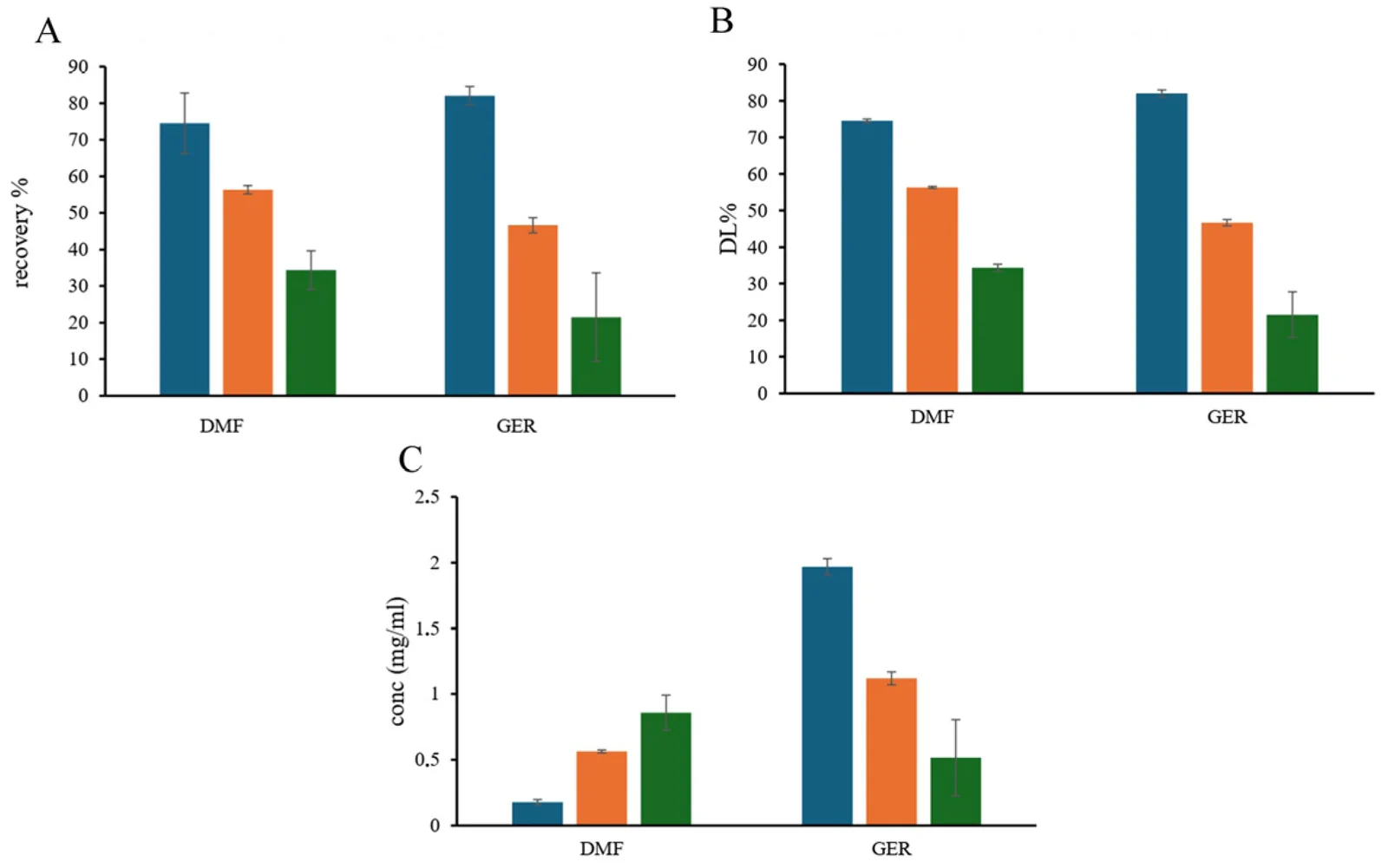
(**A**) Recovery%, (**B**) drug loading% and (**C**) total final concentration for DMF and GER in the formulation NE0.24 (DMF = 0.24 mg/mL; blue bars), NE1 (DMF = 1 mg/mL; orange bars) and NE2.5 (DMF = 2.5 mg/mL; green bars). Data are reported as mean ± SD (*n* = 3/group). Statistically significant differences for recovery (**A**), drug loading (**B**) and final concentration (**C**): DMF0.24 vs. DMF1, DMF1 vs. DMF2.5, DMF0.24 vs. DMF2.5, GER0.24 vs. GER1, GER1 vs. GER2.5, GER0.24 vs. GER2.5 (one-way ANOVA, Fisher’s post hoc test, *p* < 0.05).

Following preparation, DMF and GER recovery (Rec%), drug loading (DL%), and final concentrations were determined by HPLC. The results are reported in Figure 15. Data obtained for the formulation containing the lowest DMF concentration (NE0.24) are also included for comparison.

The results indicate that increasing the target DMF concentration in the formulation led to higher DMF recovery and drug loading, while a progressive reduction in GER recovery was observed. Since the CSOA concentration was kept constant in all formulations, these findings suggest that increasing DMF incorporation may affect the retention of GER within the NE structure. This behavior could be related to a limited capacity of the colloidal system to simultaneously accommodate increasing amounts of both compounds. Further studies investigating higher CSOA concentrations may help clarify whether the loading capacity of the system can be further improved. Despite the reduction in GER content, this compound is known to exhibit efficient transport following intranasal administration when formulated in CSOA-stabilized emulsions, likely due to the combined stabilizing and permeation-enhancing properties of CSOA [44].

Neuroprotective effects of GER have been reported in mouse models following oral administration of relatively high doses (50–200 mg/kg) [41,95]. However, pharmacokinetic studies performed in mice after oral administration of 200 mg/kg GER showed low systemic exposure, with a blood C_max_ of approximately 0.05 µg/mL and only trace amounts detected in brain tissue [96]. In contrast, oral administration of 50 mg/kg GER to rats, formulated as a coarse glycerol emulsion, resulted in markedly higher bioavailability (92%), yielding peak concentrations of approximately 280 µg/mL in blood and 2.5 µg/mL in CSF [49]. These findings highlight the strong influence of species and formulation on GER pharmacokinetics. Considering the potential advantages of the intranasal route for CNS delivery, it was considered worthwhile to investigate whether lower amounts of GER, such as those contained in the NE2.5 formulation, could still provide measurable CSF exposure following nasal administration. Based on these considerations, NE2.5 was selected for the proof-of-concept in vivo study because it provided the highest experimentally achieved DMF content while retaining GER and maintaining a nanoscale droplet size and low polydispersity. A complete biopharmaceutical and safety characterization comparable to that performed for the lower-loaded formulation was not carried out for NE2.5 and should therefore be considered a limitation of the present study, although the concentration of the stabilizing amphiphilic chitosan was the same as in the previously tested formulations, and the dimensions of the NE were comparable with those previously obtained with the selected preparation method.

Because the permeation test highlighted the importance of the NE dimensions for this aspect, before administration the NE2.5 formulation was characterized in terms of particle size (182 ± 2 nm, PDI 0.021 ± 0.027) and active contents: 1.08 ± 0.02 mg/mL DMF, 0.03 ± 0.01 mg/mL MMF and 0.28 ± 0.01 mg/mL GER. MMF appears to be derived from the degradation of DMF with an amount corresponding to ~3% of the DMF detected in the NE2.5 formulation. A similar degradation amount was recently evidenced in DMF-loaded nanoformulations designed for nasal administration [13].

#### Nasal Administration of the Formulation NE2.5

GER and DMF were administered nasally to rats with doses of 0.14 mg/kg (~0.035 mg/rat) and 0.5 mg/kg (~0.13 mg/rat), respectively, by using the NE2.5 formulation (50 μL inserted in each nostril of the rats). It is well known that GER can induce dermal and respiratory side effects, including damage to the nasal mucosa, but these effects do not occur if the amount of GER administered into the nose of rats does not exceed 20 μL [39,42]. The GER amount contained in the NE2.5 formulation administered to rats is ~0.035 mg, corresponding to ~0.04 μL, an amount ~500 times lower than the toxicity threshold in the airways of rats.

**Figure 16:**
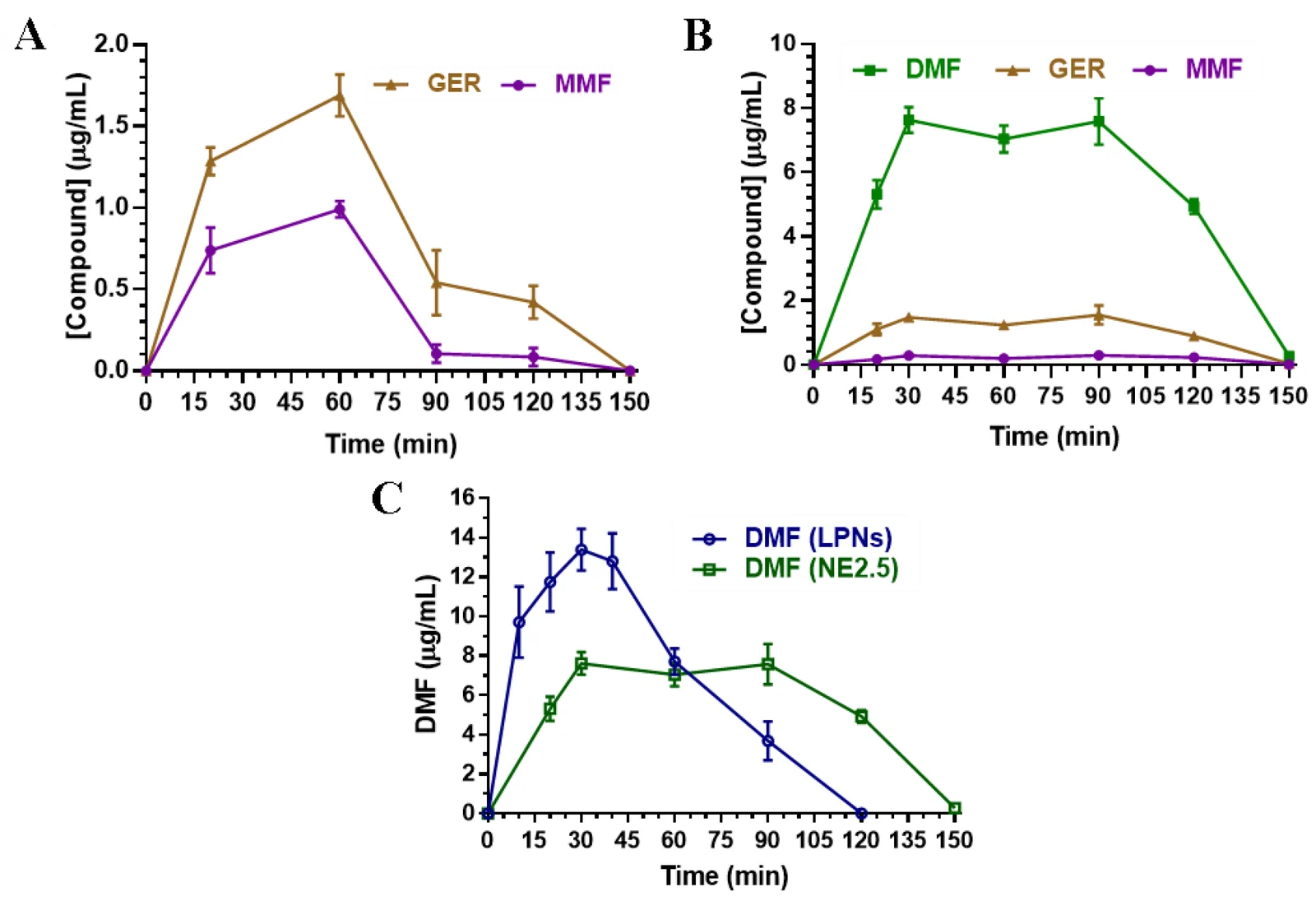
(**A**) GER and MMF profiles in the bloodstream of rats after the nasal administration of 100 μL of the NE2.5 formulation. The doses of the administered compounds are 0.14 mg/kg (~0.035 mg/rat) for GER and 0.5 mg/kg (~0.13 mg/rat) for DMF. (**B**) GER, DMF and MMF profiles in the CSF of rats after the nasal administration of 100 μL of the NE2.5 formulation. (**C**) A comparison of the DMF profiles obtained in the CSF of rats after the nasal administration of the NE2.5 formulation (0.5 mg/kg DMF dose) or a nasal formulation (1 mg/kg DMF dose) based on LPNs. All data are expressed as mean ± SD, *n* = 4).

Following intranasal administration of NE2.5, GER was detected in both the bloodstream and CSF, whereas DMF was detected in CSF but not in blood, where MMF was instead measurable (Figure 16). In our previous study, intranasal administration of free DMF at 1 mg/kg did not result in detectable DMF or MMF in CSF, whereas nanoparticle-associated DMF reached the CSF [13]. Moreover, intravenous administration of free DMF at 2 mg/kg did not result in detectable DMF in CSF [13], supporting the view that the CSF exposure observed after NE2.5 administration is unlikely to be explained simply by systemic redistribution of free DMF. Although cross-study comparisons should be interpreted cautiously, these findings support a contribution of the nanostructured formulation to DMF CSF exposure. The present study, however, does not allow the individual contributions of GER, CSOA, and the nanoemulsion architecture to be distinguished.

Figure 16A reports, in particular, the GER and MMF profiles detected in the bloodstream, showing for GER a C_max_ value of 1.69 ± 0.13 μg/mL at 60 min (T_max_) which decreased to zero within 150 min after the nasal administration; the AUC value of this profile was 126.5 ± 8.5 μg min⋅mL^−1^. The C_max_ value of MMF, detected at the same T_max_ of GER (60 min) was 0.99 ± 0.05 μg/mL and decreased to zero within 150 min; the AUC value of this profile was 62.6. ± 5.3 μg⋅min⋅mL^−1^.

A comparison between the GER profiles in the bloodstream obtained after the nasal administration of NE2.5 and an oral administration previously performed in rats with a dose of 12.5 mg (50 mg/kg) showed that this compound after oral administration was completely eliminated within 1 h [49], whereas a more prolonged GER exposure profile was observed after intranasal NE2.5 administration. This behavior seems in good agreement with the in vitro release results described above (see Section 3.7), which evidenced a maximum cumulative release of GER from NE within a few hours.

The absence of DMF in the bloodstream of rats after nasal administration of NE2.5, is not surprising considering that we have recently demonstrated that DMF is instantly hydrolyzed to MMF in the blood of rats and that, after intravenous administration of DMF to rats, only MMF can be detected in the bloodstream of rats at amounts corresponding to ~5% of the total drug administered. In particular, after the intravenous administration of 2.0 mg/kg DMF to rats, the MMF profile in the bloodstream was characterized by an AUC value of 74.7 ± 3.4 μg⋅min⋅mL^−1^ [13]. Interestingly, nasal administration of NE2.5 (0.5 mg/kg DMF) to rats was associated with an AUC value of MMF in the bloodstream of 62.6 ± 5.3 μg⋅min⋅mL^−1^ (Figure 16A). For contextual comparison, in our previous study intravenous administration of DMF (2 mg/kg) resulted in an MMF blood AUC of 74.7 ± 3.4 μg·min·mL^−1^ [13], whereas in the present study intranasal administration of NE2.5 containing 0.5 mg/kg DMF resulted in an MMF AUC of 62.6 ± 5.3 μg·min·mL^−1^. These values should not be interpreted as a formal bioavailability comparison, since MMF is a metabolite of DMF and its systemic exposure depends on route- and input-dependent absorption, hydrolysis, formation, and disposition processes. The different MMF exposure profiles may partly reflect the different input kinetics of DMF following intranasal administration of the NE and intravenous infusion, together with the rapid hydrolysis of DMF in blood.

It is important to remark that after the oral administration of DMF at therapeutic doses to humans (240 mg), this drug was absent in the bloodstream, unlike its metabolite MMF, whose C_max_ appeared at near 1 μg/mL [10], a value very similar to that obtained in the bloodstream of rats after the nasal administration of the NE2.5 formulation (Figure 16A).

Following nasal administration of NE2.5, DMF, MMF, and GER were detected in rat CSF, as reported in Figure 16B. In particular, the DMF concentration at 20 min was 5.3 ± 0.4 μg/mL and increased at 30 min to values higher than 7 μg /mL, which were maintained within 90 min, and then the DMF concentration decreased to zero within 150 min. Moreover, relatively small amounts of MMF were also detected in the CSF of rats, with concentration values ranging from ~0.2 to ~0.3 μg/mL between 20 and 120 min after the nasal administration and then decreasing to zero within 150 min (Figure 16B). The AUC values obtained from the profiles of these compounds were 823 ± 28 μg·min·mL^−1^ and 30 ± 2 μg·min·mL^−1^ for DMF and MMF, respectively, suggesting that the small amounts of MMF detected in the CSF of rats (~3% of DMF) can be attributed to the hydrolysis of DMF in the NE2.5 formulation, in accordance with a similar behavior previously evidenced for other DMF nasal formulations [13].

A potential analytical limitation associated with DMF is its susceptibility to hydrolysis to MMF in biological media. In the present study, however, freshly collected CSF samples were immediately injected into the HPLC system and were not subjected to storage or freeze–thaw cycles. Therefore, degradation during prolonged sample handling was minimized. Moreover, any hydrolysis occurring between CSF collection and chromatographic injection would be expected to decrease the measured DMF concentration while increasing MMF, rather than generating an artifactual DMF signal. This interpretation is further supported by the chromatographic separation of MMF and DMF, which eluted at 3.8 and 8.2 min, respectively.

In addition, retrospective analysis of the original HPLC-DAD data was performed to further assess the identity and chromatographic specificity of the DMF peak detected in CSF. The UV spectral profile of the peak eluting at the DMF retention time (8.2 min) in a representative CSF sample collected 30 min after NE2.5 administration closely matched that obtained from an authentic 100 μM DMF standard analyzed under the same chromatographic conditions. DAD peak-purity analysis further showed spectral homogeneity across the CSF peak, with a peak-purity index of 1.000000, exceeding the software-defined threshold of 0.999923, and no evidence of detectable co-eluting impurities under the applied chromatographic conditions (Figures S1–S5). These findings provide additional support for the assignment of the CSF peak to DMF.

Notably, DMF was readily detected in the CSF despite being undetectable in the bloodstream, where only its hydrolysis product MMF was observed. This marked dissociation between systemic and CSF profiles is consistent with the possibility that at least part of the administered DMF reaches the CSF before undergoing the rapid hydrolysis occurring in blood. This interpretation is further supported by our previous findings showing that intranasal administration of free DMF at 1 mg/kg did not result in detectable DMF or MMF in CSF, whereas DMF administered in a nanostructured formulation reached the CSF; moreover, intravenously administered free DMF was not detectable in CSF [13]. Nevertheless, CSF measurements alone cannot distinguish unequivocally between direct nose-to-brain transport and indirect routes involving systemic absorption and subsequent CNS distribution. Therefore, the present data should be interpreted as evidence of substantial and sustained DMF exposure in CSF following intranasal NE2.5 administration, with a pharmacokinetic pattern consistent with a contribution of nose-to-brain transport, rather than as a definitive demonstration of direct nose-to-brain delivery.

Figure 16C reports a comparison between two DMF profiles obtained in the CSF of rats after the nasal administration of two different formulations: the green profile is related to the NE2.5 formulation, whereas the blue profile was previously obtained by us using a nasal formulation based on lipid–polymer hybrid nanoparticles (LPNs). The DMF dose in NE2.5 was 0.5 mg/kg and produced an AUC value of 823 ± 28 μg·min·mL^−1^, very similar to the AUC value obtained by LPNs (844 ± 34 μg·min·mL^−1^), whose DMF dose was 1 mg/kg [13]. Interestingly, despite the lower DMF dose administered with NE2.5 (0.5 mg/kg) compared with the previously investigated LPN formulation (1 mg/kg), substantial and prolonged DMF concentrations were detected in CSF. In our previous study, intranasal administration of free DMF at 1 mg/kg did not result in detectable DMF or MMF in CSF, whereas DMF administered in the previously investigated LPN formulation reached the CSF. Although comparisons between separate studies should be interpreted cautiously, these previous findings support the hypothesis that formulation of DMF in nanostructured nasal delivery systems contributes to its CSF exposure.

A DMF-tocopherol acetate nanolipidic carrier has been previously shown by other authors to improve the bioavailability of MMF in both the bloodstream and brain after oral administration to rats, in comparison to the free DMF [97]. NE2.5 differs from this type of carrier, being associated with a prolonged exposure of DMF itself in the CSF, together with systemic MMF concentrations in the range reported as therapeutic in humans [10]. Considering that DMF possesses stronger immunomodulatory properties and higher membrane permeability than MMF [16,18], sustained DMF exposure in the CSF may warrant further investigation in the context of MS treatment.”

In addition to DMF and MMF, the nasal administration of the NE2.5 formulation also allowed the quantification of GER in the CSF of rats, with concentrations ranging from ~1.0 to ~1.6 μg/mL between 20 and 120 min, as reported in Figure 16B. Interestingly, these values have the same order of magnitude as GER concentrations detected in the CSF of rats after receiving an oral dose of 50 mg/kg [49], which is normally used to study the in vivo neuroprotective effects of this compound [41,96]. In particular, the C_max_ value of GER in the CSF of rats, related to this oral dose, was ~2.5 μg/mL, but its concentration rapidly decreased to zero within 1 h, whereas GER remained detectable in CSF for a longer period following intranasal NE2.5 administration.

### 3.12. Limitations of the Study

Several limitations of the present proof-of-concept study should be acknowledged. First, the absence of concurrent free-DMF, DMF-GER, blank-NE, and systemic-route comparator groups prevents attribution of the observed CSF exposure specifically to the NE and does not allow formal quantification of the relative contribution of direct nose-to-brain transport. Relevant intravenous and intranasal free-DMF data obtained under comparable experimental conditions were available from our previous study and provide useful contextual support, but they cannot replace concurrent randomized comparator groups. Second, drug concentrations were determined in CSF but not in individual brain regions; therefore, CSF exposure should not be considered equivalent to direct evidence of drug distribution within brain parenchyma. Third, although NE2.5 was characterized immediately before administration with respect to droplet size, polydispersity, and actual DMF, MMF, and GER content, it was not subjected to the complete set of in vitro biopharmaceutical and local safety studies performed during formulation optimization. In particular, direct mucin-binding, mucosal-retention, ciliotoxicity, and nasal-histopathology assessments were not performed. Fourth, the DPPH assay provides information on chemical radical-scavenging activity but does not demonstrate pharmacological synergy between DMF and GER. In addition, the cytotoxicity data showed concentration- and time-dependent effects at the higher DMF concentrations and therefore do not support a generalized definition of the formulation as non-cytotoxic. Regarding the in vitro permeability study, post-exposure TEER measurements, paracellular permeability markers, donor/receiver mass-balance analysis, and a dedicated 0.5% methanol vehicle control were not included. Therefore, mechanistic interpretation of the observed permeability differences is limited, and a possible contribution of the solvent to the permeability observed with free DMF cannot be excluded. Finally, the present in vivo study was pharmacokinetic in nature and did not evaluate therapeutic efficacy or safety in an experimental model of multiple sclerosis. These issues require dedicated comparative pharmacokinetic, pharmacodynamic, and local safety studies.

## 4. Conclusions

In the present study, an oil-in-water NE formulation was successfully developed and characterized as a promising delivery system for the administration of DMF and GER via the nose-to-brain route, aimed at the treatment of MS.

The formulation took advantage of the use of CSOA as an amphiphilic mucoadhesive stabilizer and absorption enhancer, which efficiently stabilized the lipophilic phase. Among the preparative methods evaluated, the spontaneous emulsification (SE) technique demonstrated superiority to the phase inversion (PI) one, yielding NEs with optimal physicochemical properties, including a small mean droplet size (~170 nm), a low polydispersity index (PDI < 0.3), and a positive zeta potential (>20 mV) that ensures long-term physical stability.

Thermal analyses supported by FT-IR confirmed the chemical compatibility between DMF and GER, highlighting a mutual stabilization effect.

In vitro characterization demonstrated a highly favorable biopharmaceutical profile: the NE provided a rapid drug release (exceeding 80% of the drug within 2 h) and exerted a distinct, dose-dependent ROS-scavenging activity due to the antioxidant properties of both DMF and GER. Furthermore, in vitro cell culture studies on human nasal epithelial RPMI 2650 cells demonstrated the high biocompatibility and non-cytotoxic nature of the formulation, together with good mucosal permeability, which depended strictly on the small size of the droplets.

The proof-of-concept in vivo study showed that intranasal administration of NE2.5 resulted in measurable and relatively sustained exposure of both DMF and GER in rat CSF. Notably, DMF reached concentrations of approximately 8 μg/mL in the CSF while remaining undetectable in the bloodstream, where its hydrolysis product MMF was instead observed. This distinct CSF/systemic profile, together with our previously published findings showing that free DMF administered intravenously did not reach detectable levels in CSF and that intranasal free DMF did not produce detectable DMF or MMF in CSF, supports the possibility that intranasal formulation contributes to DMF access to the CSF before extensive systemic hydrolysis. However, because the present study did not include concurrent free-drug, blank-formulation, or systemic-route comparator groups, and drug concentrations were not determined in brain tissue, the relative contribution of direct nose-to-brain transport cannot be established from the present data. Accordingly, these findings should be considered pharmacokinetic proof-of-concept evidence supporting further investigation of NE2.5 rather than a definitive demonstration of BBB bypass, direct nose-to-brain transport, or therapeutic efficacy.

## Data Availability

The original contributions presented in this study are included in the article. Further inquiries can be directed to the corresponding author.

## Supplementary Materials

The following supporting information can be downloaded at: https://www.mdpi.com/article/10.3390/pharmaceutics18091168/s1, Figure S1: Representative chromatogram recorded at 216 nm of a 100 µM (14.41 µg/mL) DMF standard solution in CSF simulation fluid constituted by a balanced Dulbecco’s Phosphate-Buffered Saline (DPBS) solution depleted of calcium and magnesium and supplemented with 0.45 mg/mL BSA; Figure S2: Representative UV spectrum of the DMF peak (R_t_ 8.2 min) following the injection in the HPLC-DAD system of a 100 µM (14.41 µg/mL) DMF standard solution in CSF simulation fluid constituted by a balanced Dulbecco’s Phosphate-Buffered Saline (DPBS) solution depleted of calcium and magnesium and supplemented with 0.45 mg/mL BSA; Figure S3: Representative chromatogram recorded at 216 nm of a CSF sample collected 30 min after nasal administration of the NE2.5 formulation; Figure S4: Representative UV spectrum of the DMF peak (R_t_ 8.2 min) following the injection in the HPLC-DAD system of a CSF sample collected 30 min after nasal administration of the NE2.5 formulation; Figure S5: Peak purity of the DMF peak (R_t_ 8.2 min) following the injection in the HPLC-DAD system of a CSF sample collected 30 min after nasal administration of the NE2.5 formulation. The purity curve (black) remains aligned with the zero line (red) across the entire peak width. No impurities were detected, confirmed by a peak impurity index of 1.000000 (single-point threshold: 0.999923) and a minimum peak purity index of 76, indicating spectral homogeneity of the chromatographic peak and no detectable evidence of co-eluting impurities under the applied HPLC-DAD conditions.
