# Supplementary material for: Mucoadhesive Nanoemulsions for Nose-to-Brain Delivery of Dimethyl Fumarate: Development, Characterization and Proof-of-Concept In Vivo Evaluation
Source: Pharmaceutics. 2026 Sep 16;18(9):1168. doi: 10.3390/pharmaceutics18091168 (PMC13610156; doi:10.3390/pharmaceutics18091168)
Supplement: Supplementary file 1 [file pharmaceutics-18-01168-s001.zip › pharmaceutics-4495422-supplementary.pdf]

Supplementary Materials

Mucoadhesive Nanoemulsions for Nose-to-Brain Delivery of Dimethyl Fumarate: Development, Characterization and Proof-of-Concept In Vivo Evaluation

Eleonora Sofia Cama, Giada Botti, Sara Perteghella, Laura Catenacci, Fahad Khan Tareen, Sarah Beggiato, Maria Cristina Bonferoni, Luca Ferraro, Milena Sorrenti, Alessandro Dalpiaz

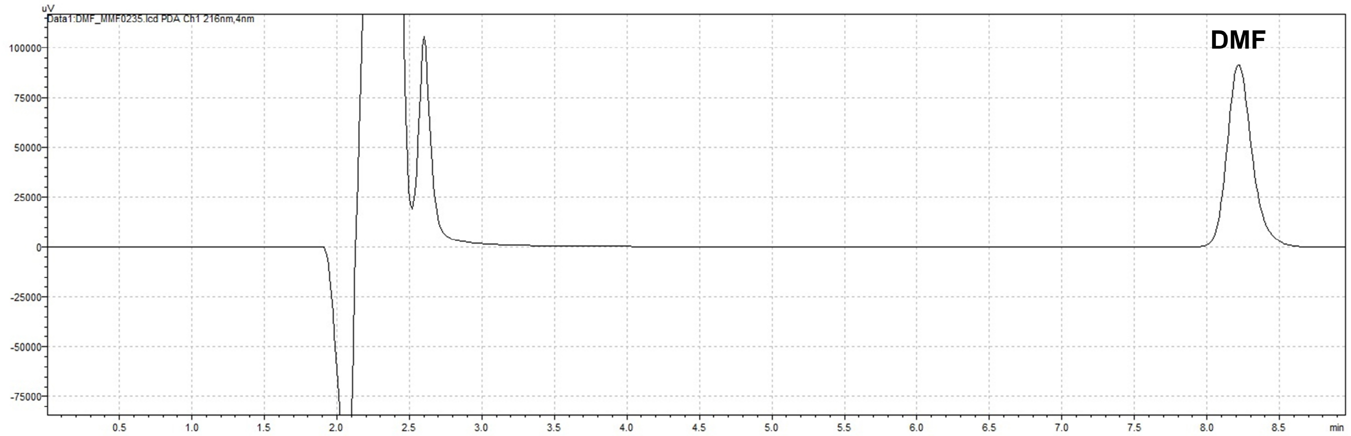

Figure S1. Representative chromatogram recorded at 216 nm of a 100 µM (14.41 µg/mL) DMF standard solution in CSF simulation fluid constituted by a balanced Dulbecco's Phosphate-Buffered Saline (DPBS) solution depleted of calcium and magnesium and supplemented with 0.45 mg/mL BSA.

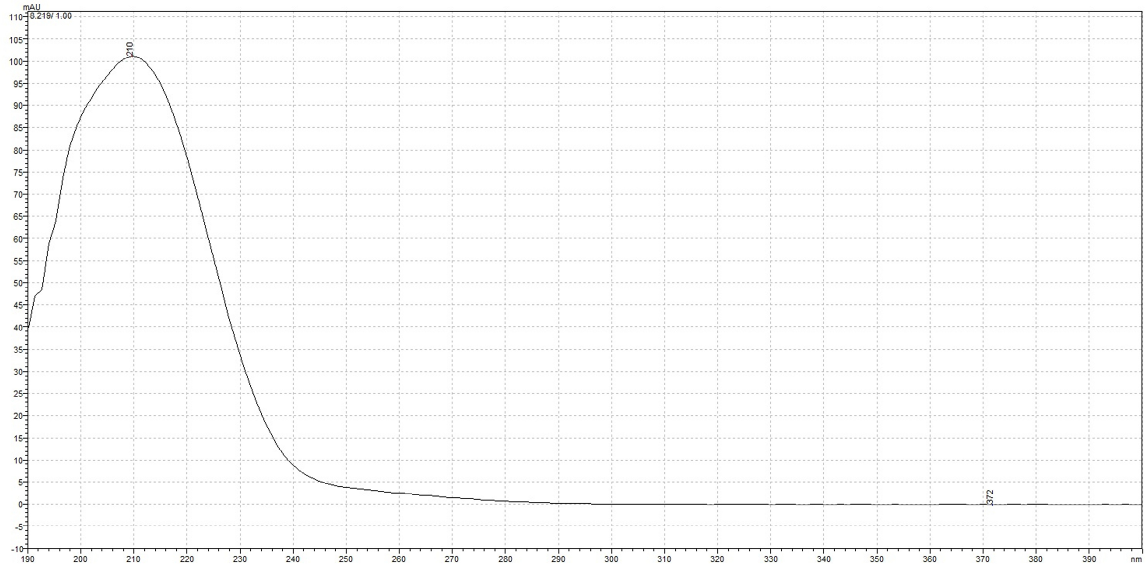

Figure S2. Representative UV spectrum of the DMF peak (R_t 8.2 min) following the injection in the HPLC-DAD system of a 100 µM (14.41 µg/mL) DMF standard solution in CSF simulation fluid constituted by a balanced Dulbecco's Phosphate-Buffered Saline (DPBS) solution depleted of calcium and magnesium and supplemented with 0.45 mg/mL BSA.

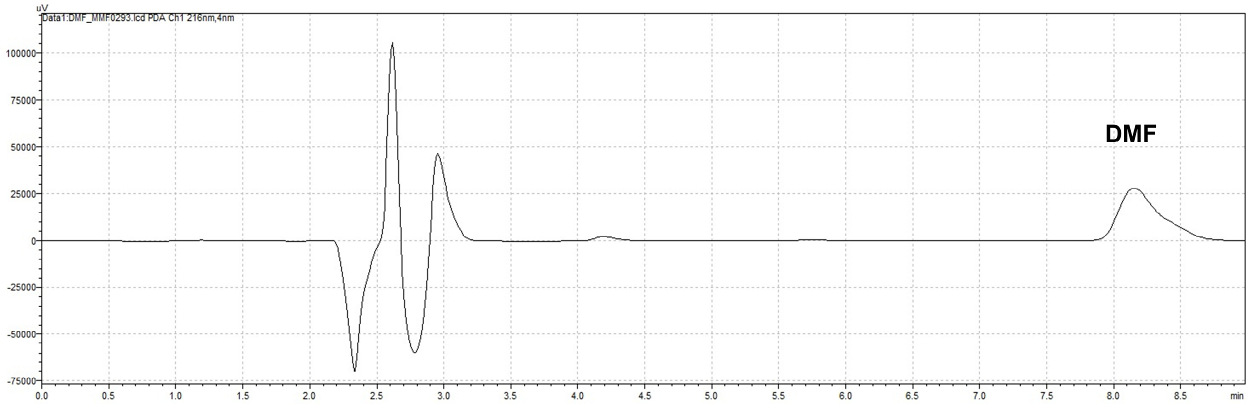

Figure S3. Representative chromatogram recorded at 216 nm of a CSF sample collected 30 min after nasal administration of the NE2.5 formulation.

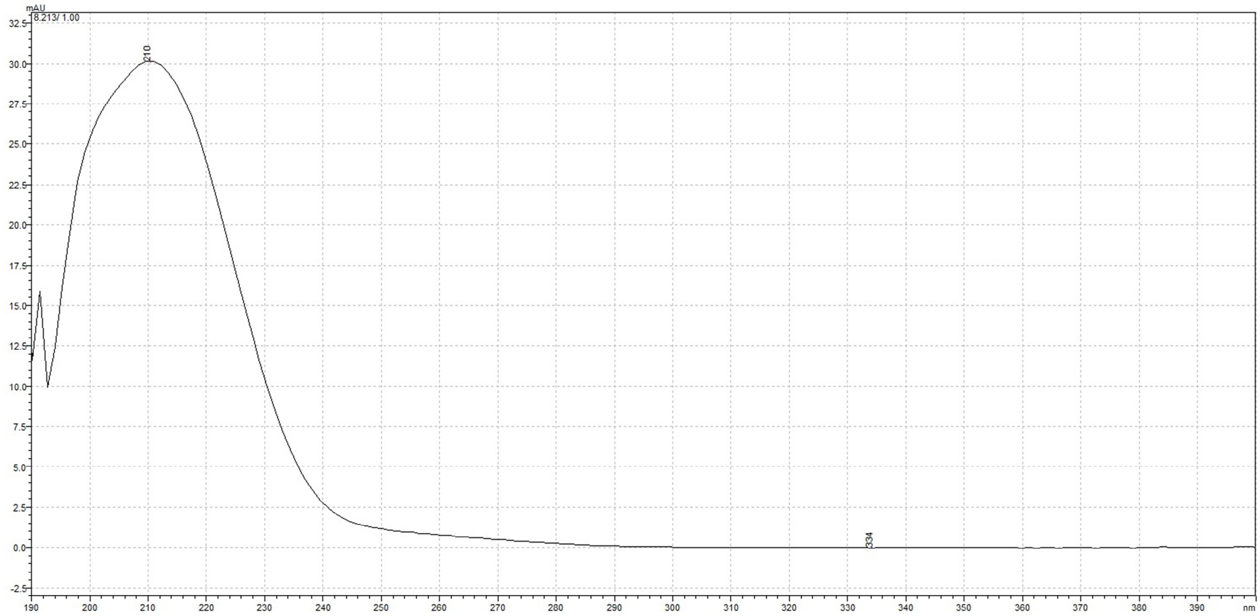

Figure S4. Representative UV spectrum of the DMF peak (R_t 8.2 min) following the injection in the HPLC-DAD system of a CSF sample collected 30 min after nasal administration of the NE2.5 formulation.

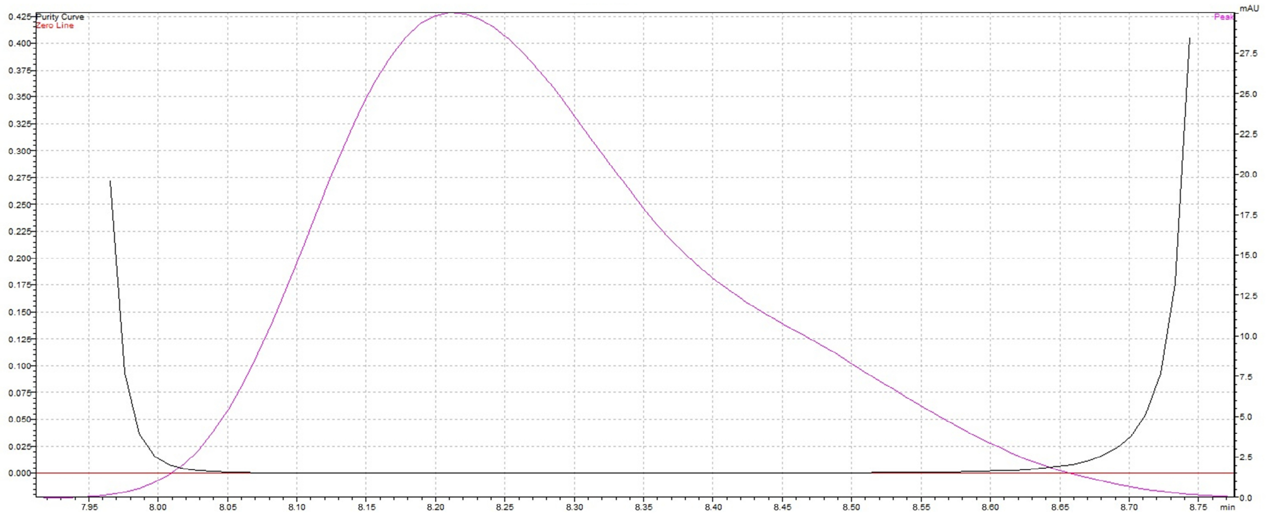

Figure S5. Peak purity of the DMF peak (R_t 8.2 min) following the injection in the HPLC-DAD system of a CSF sample collected 30 min after nasal administration of the NE2.5 formulation. The purity curve (black) remains aligned with the zero line (red) across the entire peak width. No impurities were detected, confirmed by a peak impurity index of 1.000000 (single-point threshold: 0.999923) and a minimum peak purity index of 76, indicating spectral homogeneity of the chromatographic peak and no detectable evidence of co-eluting impurities under the applied HPLC-DAD conditions.
